# Establishment of a tumor immune microenvironment-based molecular classification system of breast cancer for immunotherapy

**DOI:** 10.18632/aging.203682

**Published:** 2021-11-11

**Authors:** Xiaobo Zheng, Li Li, Chune Yu, Jiqiao Yang, Yujie Zhao, Chao Su, Jing Yu, Mingqing Xu

**Affiliations:** 1 Department of Liver Surgery, West China Hospital, Sichuan University, Chengdu, Sichuan 610041, China; 2 Laboratory of Tumor Targeted and Immune Therapy, Clinical Research Center for Breast Disease, West China Hospital, Sichuan University, Chengdu, Sichuan 610041, China; 3 Institute of Clinical Pathology, West China Hospital, Sichuan University, Chengdu, Sichuan 610041, China; 4 Department of Hepatopancreatobiliary Surgery, Meishan City People’s Hospital, Meishan Hospital of West China Hospital, Sichuan University, Meishan, Sichuan 610020, China

**Keywords:** breast cancer, immune-related subtype, tumor microenvironment, cancer immunotherapy, non-negative matrix factorization

## Abstract

Antitumor immunotherapy can enable promising and durable responses following their clinical application. However, heterogeneity in the tumor immune microenvironment leads to differences in the individual response rates. In this study, we identified novel immune-related molecular subclasses of breast cancer using a non-negative matrix factorization analysis. We enrolled 4184 patients with breast cancer, including 1104 patients from The Cancer Genome Atlas as a training cohort and 3080 patients from another four independent datasets as validation cohorts. In the training cohort, 36.9% of patients who exhibited significantly higher immunocyte infiltration and enrichment of immune response-associated signatures were categorized into an immune class, which was confirmed by probing the expression of immunocyte markers (CD3, CD19, and CD163). Within the immune class, 53.3% of patients belonged to an immune-suppressed subclass, characterized by the activation of stroma-related signatures and immune-suppressive cells. The remaining patients in the immune class were allocated to an immune-activated subclass. The interferon-γ and granzyme B levels were higher in the immune-activated subclass, whereas the transforming growth factor-β1 and programmed cell death-1 (PD-1) levels were higher in the immune-suppressed subclass. The established molecular classification system was recapitulated in validation cohorts. The immune-activated subclass was predicted to have a better response to anti-PD-1 immunotherapy. The immune-related subclasses were associated with differences in copy number alterations, tumor mutation burden, neoantigens, tumor-infiltrating lymphocyte enrichment, PD-1/programmed death-ligand 1 expression, mutation landscape, and various infiltration immunocytes. Overall, we established a novel immune-related molecular classification of breast cancer, which may be used to select candidate patients for immunotherapy.

## INTRODUCTION

Breast cancer is the most common malignancy in women worldwide, with a continuous increase in its incidence [1]. Over the past decades, advancements in determining the molecular mechanisms of breast cancer have led to the identification of canonical markers for breast cancer subtypes, including immunohistochemical, proliferative, genomic, and immune markers [2]. Additionally, endocrine therapy, targeted therapy, chemotherapy, and immunotherapy have been preferably used for individual breast cancer subtypes [3, 4]. For example, neoadjuvant combination therapy, which adopts targeted agents and chemotherapy, is recommended for human epidermal growth factor receptor 2 (HER2)-positive and triple-negative breast cancers [5]. For advanced breast cancer with metastasis, the use of small molecule inhibitors and immunotherapy are fundamental strategies based on the tumor subtype and molecular characteristics [6]. Despite improvement in the oncologic outcomes of patients with breast cancer in recent years, most patients with advanced breast cancer are at a higher risk of relapse and distant metastasis, which ultimately leads to their death [7, 8]. Traditional classification systems and prognostic prediction markers do not accurately reflect the biological heterogeneity and clinical complexity of breast cancer. Therefore, it is important to identify novel molecular subclasses contributing to tumor heterogeneity to guide optimal clinical management.

Antitumor immunotherapy, which enhances immune activation, has shown encouraging results in patients with breast cancer [9, 10]. In a recent phase-III randomized controlled trial (IMpassion130), patients with breast cancer were successfully treated with programmed cell death-1 (PD-1) [11]. Combination therapy of immune checkpoint inhibitors (ICIs) with nanoparticle albumin-bound-paclitaxel has been approved as the standard first-line therapy in a subpopulation of patients with metastatic triple-negative breast cancer [12]. Additionally, the KEYNOTE-522 study suggested the use of immunotherapy in patients with early breast cancer [13]. However, our incomplete understanding of tumor microenvironment (TME) interactions limits the clinical application of immunotherapy, as only a subset of patients with breast cancer benefit from antitumor immunotherapy [14]. Therefore, further dissection of the components of the TME and identification of individual patient molecular characteristics and immune status could provide vital information for tailoring appropriate strategies for candidate patients.

Computational algorithms have been applied for the dissection of transcriptomic sequencing data for cancer subtyping. Non-negative matrix factorization (NMF) is an analysis method that could aid in virtually micro dissecting the molecular characteristics from bulk gene expression profile data. NMF is considered versatile in characterizing various immune landscapes in hepatocellular carcinoma and small-cell lung cancer [15, 16]. In this study, we used NMF to dissect the gene expression profiles in breast cancer. We established a novel immune-related classification system of breast cancer that could guide the selection of candidate patients for immunotherapy.

## RESULTS

### Establishment of a novel immune-related molecular classification system for patients with breast cancer

To identify the immune-related subclass of patients with breast cancer, we comprehensively dissected the mRNA expression profiles using a combinational algorithm (Supplementary Figure 1). A total of 4184 patients with breast cancer were enrolled from public databases, including 1104 patients from The Cancer Genome Atlas (TCGA; as the training cohort) and 3080 patients from four external cohorts (as the validation cohort; Table 1). Ten expression patterns were identified in the training cohort using the NMF algorithm (Supplementary Figure 2A). Using the ESTIMATE algorithm, the immune enrichment score of each patient was calculated. The average expression value of the eighth pattern was significantly higher than that of other patterns (Supplementary Figure 2B). Thus, we regarded this pattern as an “immune factor.” The maximum NMF decomposition weight among the remaining nine patterns was selected as the representative of these nine patterns, and then the genes were sorted according to the difference between weight of pattern 8 and maximum weight of the other patterns, and the top 150 genes were selected as "exemplar genes,” which are presented in Supplementary Table 1. These exemplar genes were highly enriched in signaling involved in immune activation, such as B-cell-mediated immunity, complement activation, immunoglobulin-mediated immune response, humoral immune response, immunoglobulin complex, antigen binding, and antigen processing and presentation (Supplementary Figure 3A, 3B). These results further verified immune-related signaling and functions of the immune factor.

**Table 1:** Patient characteristics.

	**TCGA**	**GSE2109**	**GSE25066**	**GSE58644**	**METABRIC**
**Overall**	1104	350	508	318	1904
Data array	RNA-seq	Microarray	Microarray	Microarray	Microarray
**Age (years)**					
≥60	514	165	104	143	1062
<60	589	184	404	175	842
**Sex**					
Male	12	–	–	–	0
Female	1091	–	–	–	1904
**Stage**					
T0		–	3	–	–
T1	281	–	30	43	–
T2	641	–	255	58	–
T3	138	–	145	13	–
T4	40	–	75	1	–
**ER status**					
Positive	814	160	297	247	1459
Negative	239	83	205	70	445
**PR status**					
Positive	704	127	243	–	1009
Negative	346	113	258	–	895
**HER2 status**					
Positive	164	61	6	58	236
Negative	566	163	485	253	1668

Abbreviations: ER: estrogen receptor; PR: progesterone receptor; HER2: human epidermal growth factor receptor 2; TCGA: The Cancer Genome Atlas.

**Figure 1:**
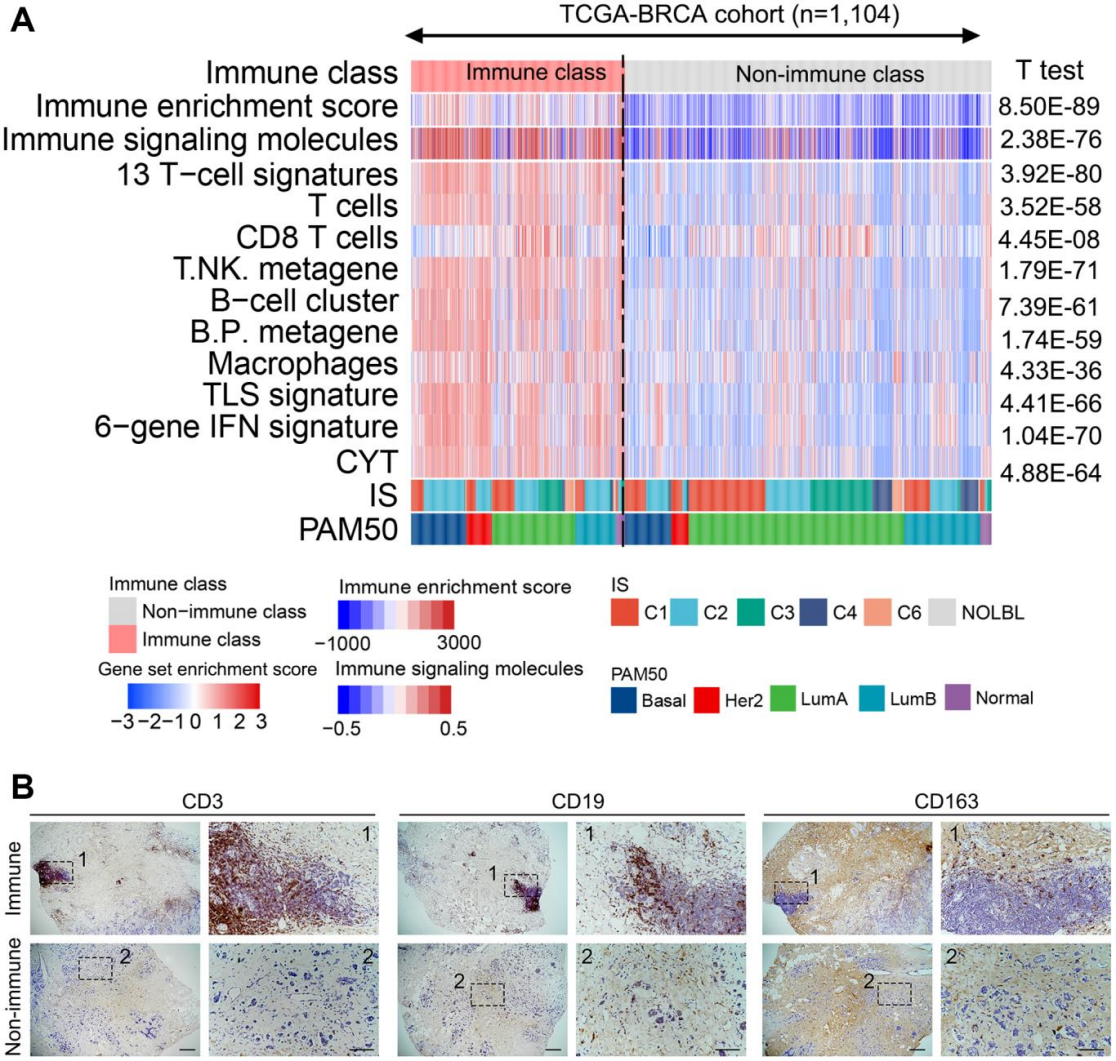
**Identified immune-related subclasses of patients with breast cancer.** (**A**) Consensus-clustered heatmap on the TCGA-BRCA cohort (*n* = 1,104) based on the example genes of immune factors selected by NMF, and refine it through the multidimensional scaling random forest to define the immune class (407/1104, 36.9%, pink bar). Immune-related signatures were compared between immune and non-immune classes. Red and blue bars correspond to high and low scores of each signature, respectively. (**B**) Representative images of CD3, CD19, and CD163 (markers of T, B, and myeloid cells, respectively) staining in the immune and non-immune classes. Scale bar, 100 μm. Abbreviations: IFN: interferon; TLS: tertiary lymphoid structure; CYT: cytolytic activity score; NMF: non-negative matrix factorization; TCGA–BRCA: The Cancer Genome Atlas-Breast Cancer.

Based on the 150 example genes, the patients in the training cohort were roughly classified by consensus clustering, which was refined using the multidimensional scaling random forest algorithm to group the patients more precisely (Supplementary Figure 4A). Two groups were established, one accounted for 36.9% of patients in the training cohort (407/1104) and the other accounted for 63.1% of patients (697/1104) (Supplementary Figure 4B). The previously established immune-related signatures were analyzed to explore the characteristics of the established subclasses (Supplementary Table 2). The immunocyte-related signatures, tertiary lymphoid structures (TLS), IFN signatures, and cytolytic activity (CYT) scores of patients in the high-immune enrichment group were significantly higher than those of patients in the low-enrichment group (all *P* < 0.05, Figure 1A). This high-immune enrichment group was considered as the immune class, whereas the low-enrichment group was defined as the non-immune class. These results were confirmed in our clinical tumor samples via immunohistochemistry staining. The immune and non-immune classes were screened from our collected tumor samples with RNA sequencing data using the above-mentioned algorithm. The levels of CD3, CD19, and CD163 (markers of T, B, and myeloid cells) in the immune class were higher than those in the non-immune class (Figure 1B). Moreover, the functional enrichment analysis revealed that differentially expressed genes (DEGs) between the immune and non-immune classes were mainly associated with immune-related pathways, including Th17/Th1/Th2 cell differentiation, complement activation, adaptive immune response, and lymphocyte-mediated immunity (Supplementary Figure 5A, 5B). Similarly, immune cells and immune response-related pathways were significantly activated in the immune class, as determined using the gene set enrichment analysis (Supplementary Figure 5C).

The molecular classes were compared with those in previously reported breast cancer-related molecular classification to further validate the robustness of the immune-based classification system. Thorsson et al. [17] identified six subclasses widely used for immune-related classification. PAM50, a 50-gene signature, provides intrinsic molecular subclasses for risk stratification in patients with breast cancer [18]. IFN-γ dominant (204/407 vs. 191/697, *P* < 0.01) and ER^-^ (HER2 and Basal, 154/407 vs. 121/697, *P* < 0.01) subtypes were more enriched in the immune class, whereas wound healing (108/407 vs. 265/697, *P* < 0.01) and ER^+^ (LumA and LumB, 235/407 vs. 554/697, *P* < 0.01) subtypes were significantly reduced (Figure 1A). The IFN-γ dominant and ER^-^ subtypes are reportedly associated with high immunogenicity, implying the immune activation characteristics of the immune class.

### Immune class was divided into immune-activated and immune-suppressed subclasses based on the activation of stromal signatures

**Figure 2:**
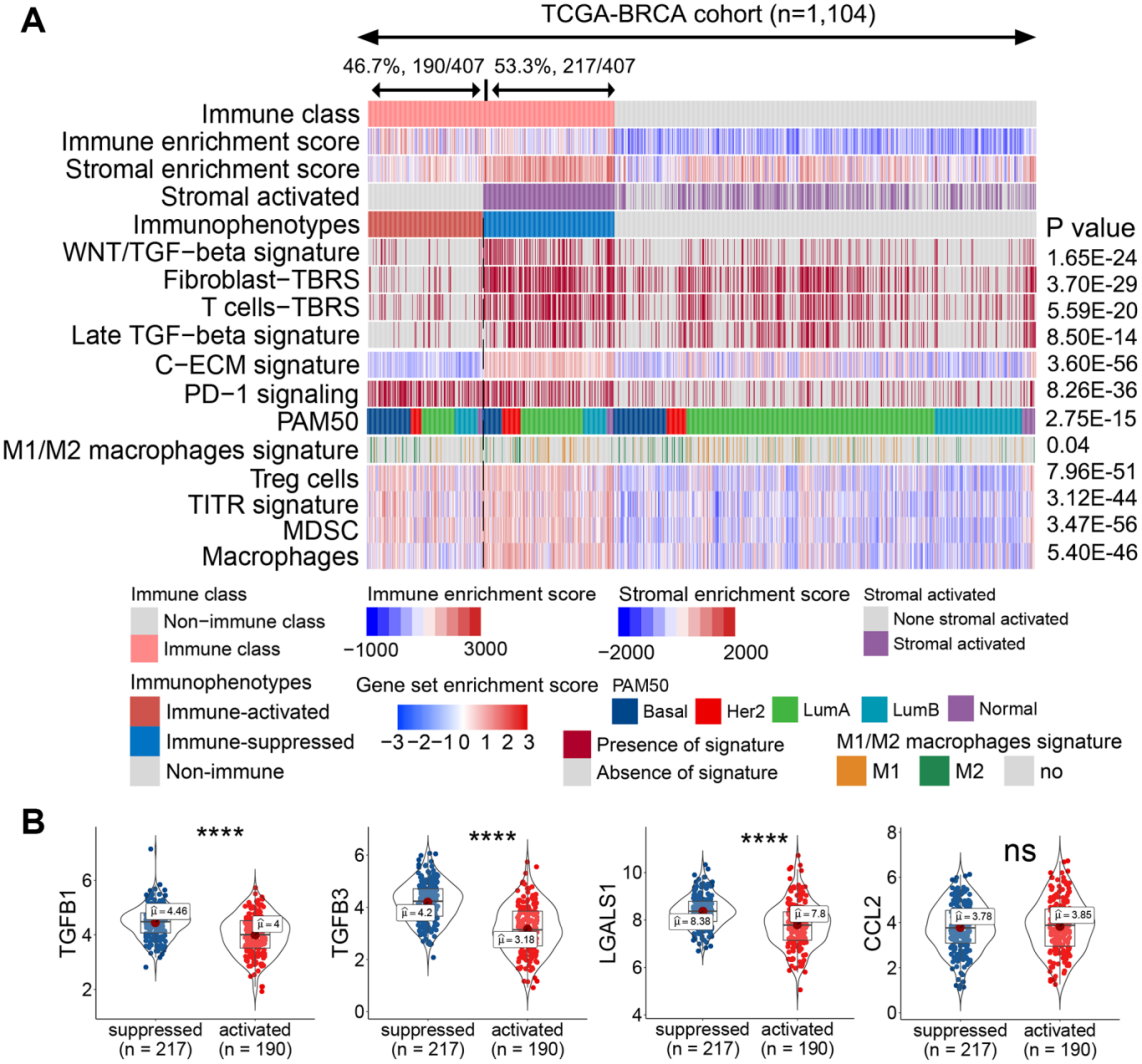
**Identified immune-activated and -suppressed subclasses of the immune class.** (**A**) Based on the activation of stromal signatures, the immune class was divided into immune-suppressed (217/407, 53.3%; blue bar) and immune-activated (190/407, 46.7%, red bar) subclasses by NTP. Immune suppression-related signatures were compared between the immune-activated and -suppressed classes. Red and blue bars correspond to high and low scores of each signature, respectively. The signatures predicted to be positive by the NTP algorithm are marked in red, and those predicted to be negative are marked in gray. (**B**) Expression levels of immunosuppressive genes were compared between the immune-activated and -suppressed subclasses. Abbreviations: TITR: tumor-infiltrating Tregs; MDSC: myeloid-derived suppressor cell; C-ECM: cancer-associated extracellular matrix; NTP: nearest template prediction; ns: not significant. *P* > 0.05, ^****^P ≤ 0.0001).

The role of the TME component in regulating immune activation varies with the release of chemokines, cytokines, and other soluble factors, as well as ligands expressing inhibitory receptors [19, 20]. To explore the component influencing the immunotherapeutic effect, the immune class was further analyzed using the nearest template prediction (NTP) algorithm. As shown in Figure 2A, 46.7% (190/407) of patients showed a lack of stromal-activated signatures and relatively low stromal enrichment scores in the immune class, whereas the remaining 53.3% (217/407) of patients in the immune class presented the opposite results. Compared with patients lacking stromal-activated signatures, patients with stromal-activated signatures showed higher TGF-β signatures, such as the Wnt/TGF-β, fibroblast TGF-β response (fibroblast-TBR), T cell TGF-β response (T cell-TBR), late TGF-β, and cancer-associated ECM (C-ECM) signatures (Figure 2A). Pinyol et al. reported a Wnt/TGF-β proliferation subclass with immunosuppressive pro-carcinogenic microenvironment [21]. TGF-β suppresses CD4^+^ T helper 2 cell-mediated anti-cancer immunity, and blocks TGF-β signaling prevents breast cancer progression [22]. TGF-β is a recognized immunosuppressant in the immune microenvironment, and C-ECM regulated by activated CAFs can also recruit immunosuppressive cells [23, 24]. Therefore, we defined the stromal-activated group as the immune-suppressed subclass, whereas patients with a lack of stromal activation were included in the immune-activated subclass. Additionally, the immunosuppressive cell-related signature (i.e., Treg cells, tumor-infiltrating Treg cells (TITRs), myeloid-derived suppressor cells (MDSCs)) and PD-1 signaling were upregulated in the immune-suppressed subclass compared with those in the immune-activated subclass (Figure 2A). The mRNA expression of immunosuppressive genes such as *TGFB1*, *TGFB3*, and *LGALS1* was also significantly upregulated in the immune-suppressed class (Figure 2B).

**Figure 3:**
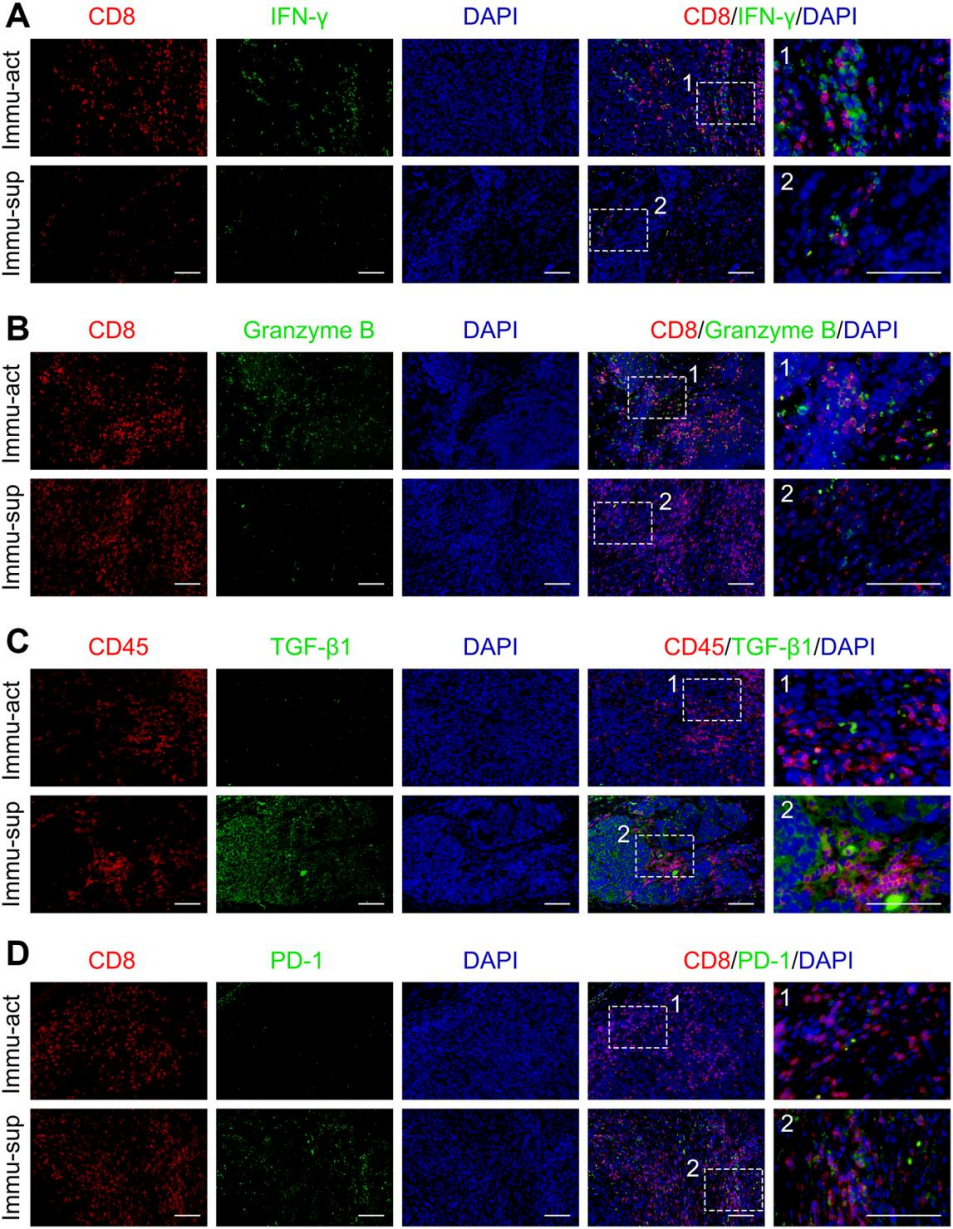
**Immune-activated and -suppressed status of the tumor niche.** (**A**) Co-staining of CD8 and IFN-γ in immune-activated and -suppressed patients. (**B**) Co-staining of CD8 and granzyme B in immune-activated and -suppressed patient samples. (**C**) Co-staining of CD45 and TGF-β1 in immune-activated and -suppressed patient samples. (**D**) Co-staining of CD8 and PD-1 in immune-activated and -suppressed patient samples. Scale bar, 100 μm. Abbreviations: Immu-act: immune-activated; Immu-sup: immune-suppressed.

To validate the varied TME status, we probed the protein levels of markers representing immune activation and suppression in our collected clinical tumor samples by immunofluorescence staining. The immune-activated and -suppressed subclasses were screened from our collected tumor samples with RNA sequencing data using the NTP algorithm. The protein levels of IFN-γ and granzyme B co-staining with CD8^+^ T cells were upregulated in the immune-activated patients compared with those in the immune-suppressed patients (Figure 3A, 3B). Notably, the TGF-β1^+^CD45^+^ or PD-1^+^CD8^+^ double-positive cells were higher in the immune-suppressed patients than in the immune-activated patients (Figure 3C, 3D). Collectively, the immune class could be further subdivided according to the component of TME, implicating that immune-suppressive signaling and immune-suppressive cell elimination could potentially improve anti-cancer immunotherapy in patients with immune-suppressed status.

### Recurrence of the established molecular classification in four independent cohorts

To recapitulate our established immune-based molecular classification, four additional external cohorts with mRNA expression profiles (i.e., METABRIC, GSE2109, GSE5066, and GSE58644) were examined. Based on the top 150 DEGs defined as a classifier, patients in the validation cohorts were separated into immune and non-immune classes; the immune class was further subdivided into immune-activated and -suppressed subclasses as mentioned for the training cohort. In METABRIC, 48.6% (926/1904) of patients with higher enrichment scores for immune-related signatures were allocated to the immune class, whereas the remaining 51.4% (978/1904) of patients were allocated to the non-immune class. In the immune class, 56.6% (524/926) of patients who showed a lack of activated tumor-stromal characteristics were assigned to the immune-activated subclass (Supplementary Figure 6). Notably, these three immune-related subclasses were recapitulated in the other three validation cohorts (Supplementary Figures 7–9). The proportion of patients in the immune class was approximately 50%, and that in the immune-activated subclass was 50.3%–64.5% in all cohorts. Consistently, immune enrichment scores, immune signaling molecules, and immune-related signatures of the immune class were significantly upregulated compared with those in the non-immune class. Signatures related to immune suppression (e.g., TGF-β-related signatures, Treg cells, TITR, and MDSCs) were significantly higher in the immune-suppressed subclass than in the immune-activated subclass. The mRNA expression of immunosuppressive genes such as *TGFB3* and *LGALS1* was also significantly upregulated in the immune-suppressed subclass compared with that in the immune-activated subclass (Supplementary Figure 10). Collectively, the use of NMF consensus clustering and NTP could accurately and robustly classify patients with breast cancer into three types of immunophenotypes.

### Identified molecular subclasses associated with response to immunotherapy

**Figure 4:**
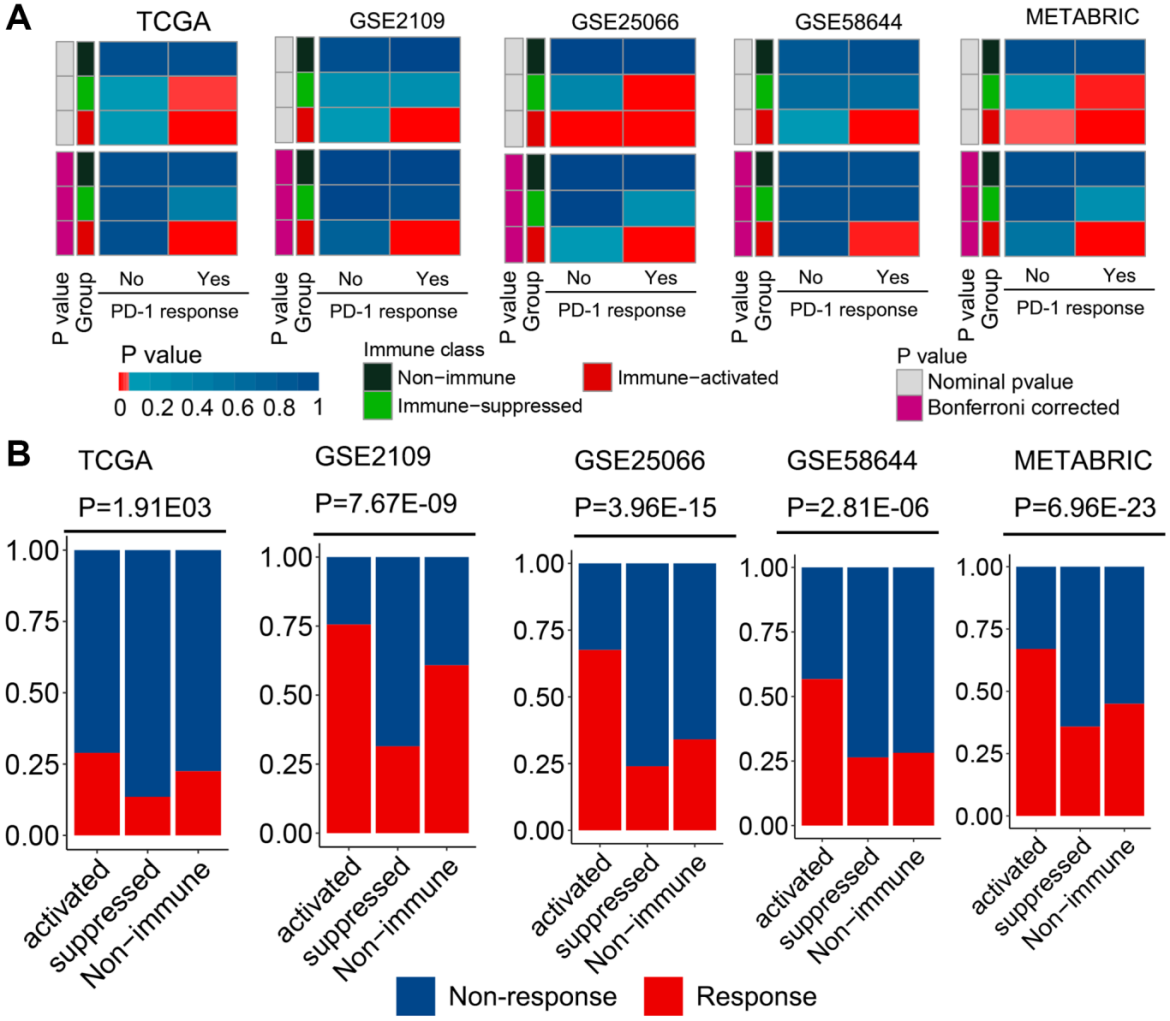
**Different responses to immunotherapy of patients belonging to the established molecular subclasses.** (**A**) Similarity between patients of different subclasses and patients with melanoma who received ICI treatment were compared using the submap algorithm. Patients in the immune-activated subclass had higher mRNA expression profile similarity with patients that responded to anti-PD-1 treatment. (**B**) Distribution of the clinical response to ICI treatment in the three immunophenotypes was determined using the TIDE analysis. Patients in the immune-activated subclass had a higher predicted response rate than the other two groups.

For a newly defined immunophenotype, its guidance in clinical treatment is a concern. Therefore, the potential of our immunophenotype classification system to predict ICI treatment response was evaluated in all cohorts. The mRNA expression profile similarity between patients with breast cancer and those with melanoma receiving anti-PD-1 treatment was calculated using a submap algorithm. Notably, patients in the immune-activated subclass presented higher mRNA expression profile similarity with those who responded to anti-PD-1 treatment than patients in the other two groups (Figure 4A). These results indicate that patients in this subclass can gain more benefits from anti-PD-1 immunotherapy. Tumor immune dysfunction and exclusion (TIDE) is a well-accepted algorithm for evaluating a patient’s response to ICI treatment based on tumor expression profile data [25]. The results of TIDE revealed that patients in the immune-activated subclass had a higher predicted response rate than those in the other two groups (Figure 4B). Collectively, these results indicated that patients in the immune-activated subtype could be candidate patients for receiving ICI treatment.

### Heterogeneity in tumor molecular characteristics between immune-related subclasses

**Figure 5:**
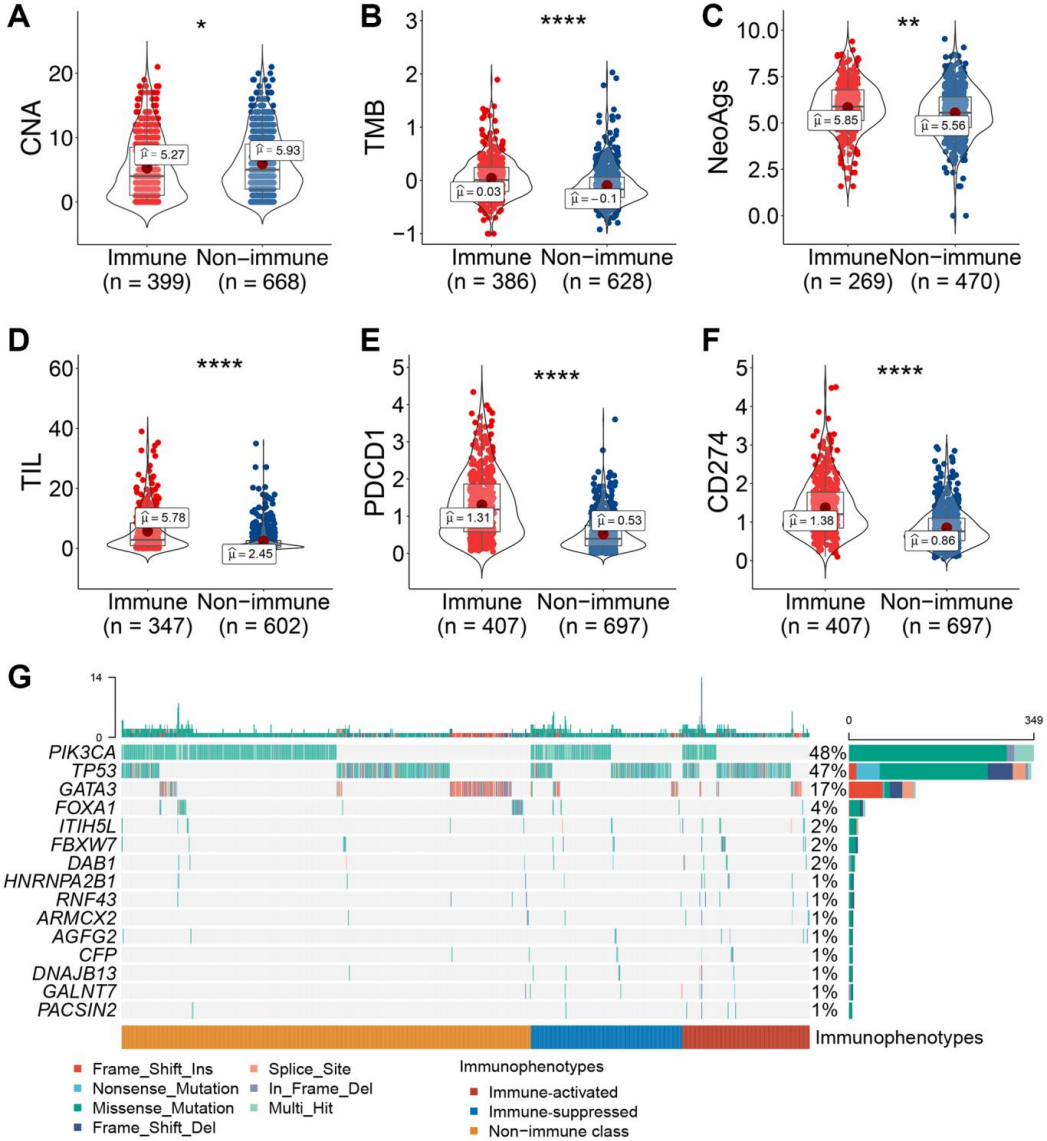
**Association between immune-related molecular subclasses and molecular characteristics.** (**A**–**F**) Copy number deletion at the arm level (**A**), TMB (**B**), neoantigens (**C**), TIL abundance (**D**), and PD-1/PD-L1 mRNA expression levels (**E**, **F**) were compared between patients in the immune and non-immune subclasses. (**G**) Oncoprint of differentially mutated tumor-related genes among the three immunophenotypes. Abbreviations: TIL: tumor-infiltrating lymphocytes; TMB: tumor mutant burden; NeoAg: neoantigen. ^*^P ≤ 0.05, ^**^P ≤ 0.01, ^***^P ≤ 0.001, or ^****^P ≤ 0.0001.

To explore the molecular mechanism leading to varied immunophenotypes, a series of tumor molecular characteristics was compared between immune-related subclasses. Patients in the immune class had a lower deletion burden of copy number alterations (CNAs) at the arm level than those in the non-immune class (Figure 5A). Additionally, the immune class had a higher level of tumor mutation burden (TMB), neoantigens (NeoAgs), tumor-infiltrating lymphocytes (TILs), and PD-1/PD-L1 expression than the non-immune class (Figure 5B–5F). The mutation landscape of the three immunophenotypes showed an obvious discrepancy in the MutSigCV algorithm. *TP53* mutation frequency was significantly higher in the immune class than in the non-immune class (immune class, 47.7% vs. non-immune class, 15.4%, *P* < 0.01) (Figure 5G). In the immune-activated subclass, the mutation percentage of *ITIH5L* and *FBXW7* was significantly higher than that in the other two subclasses (*ITIH5L*: immune-activated, 4.4% vs. immune-suppressed, 2.0% and non-immune, 0.96%; *FBXW7*: immune-activated, 4.4% vs. immune-suppressed, 2.9% and non-immune, 0.80%, respectively, all *P* < 0.05). *PIK3CA* showed a higher mutation percentage in the immune-suppressed subclass than that in the other two subclasses (immune-suppressed, 41.5% vs. immune-activated, 20.0% and non-immune, 36.3%, respectively, *P* < 0.01). In the non-immune class, the frequency of *GATA3* mutation was significantly upregulated (non-immune, 14.5% vs. immune-activated, 9.4% and immune-suppressed, 8.8%, respectively, *P* = 0.04). Collectively, the immune class had higher TMB, NeoAgs, TIL enrichment, and PD-1/PD-L1 expression and lower CNA, implying that all these factors underlie the mechanism of varied immunophenotypes of breast cancer.

### Infiltrated immune cell types correlated with immune-related subclasses

**Figure 6:**
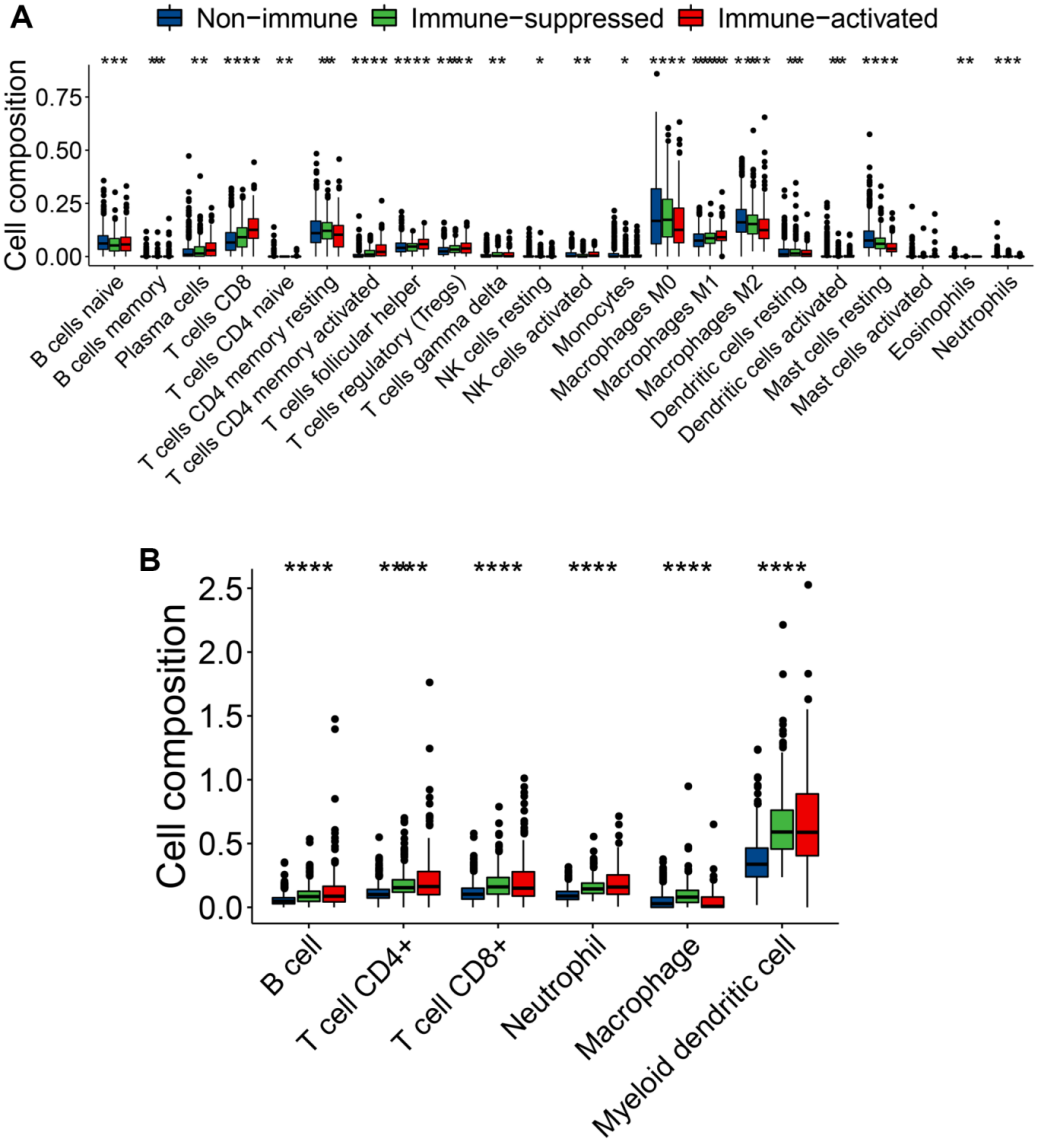
**Varied infiltrating immune cell subtypes among the three immunophenotypes.** (**A**, **B**) Comparison of the immune cell type composition among the three immunophenotypes using CIBERSORT (**A**) and TIMER (**B**) algorithms. ns (not significant): *P* > 0.05, ^*^P ≤ 0.05, ^**^P ≤ 0.01, ^***^P ≤ 0.001, or ^****^P ≤ 0.0001, ns symbol was hidden.

Different immune cell subtypes may have antitumor or protumor effects. For example, CD8^+^ cytotoxic T cells, natural killer cells, CD4^+^ T helper cells, and M1 macrophages have antitumor roles, whereas Treg cells, M2 macrophages, and MDSCs assist in tumor immune evasion. To explore the variations in the immune cell components among the three immunophenotypes, we compared constituent ratio of multiple immune cell subtypes in the training set. Using CIBERSORT analysis, the ratio of cytotoxic immunocytes (such as plasma cells, CD8^+^ T cells, memory-activated CD4^+^ T cells, follicular helper T cells, and M1 macrophages) was significantly higher in the immune-activated subclass, implying immune activation status (Figure 6A). In contrast, the ratio of memory resting CD4^+^ T cells, M0 macrophages, M2 macrophages and resting mast cells was significantly higher in the immune-suppressed subclass compare with immune-activated subclass (Figure 6A). Using the TIMER algorithm, the ratio of CD4^+^ T cells and neutrophils was slightly higher in the immune-activated subclass, whereas the ratio of macrophages was significantly higher in the immune-suppressed subclass (Figure 6B). These results further confirmed the immune-activated and -suppressed status of the patients with breast cancer.

## DISCUSSION

Although immunotherapy with ICIs shows promising results in anti-cancer therapy, only a small subset of patients responds to it [26]. Exploring predictive markers and stratifying patients based on certain characteristics are important strategies to improve the effect of immunotherapy [27]. Although classic molecular classification systems of breast cancer already exist, they do not account for the immune status and cannot effectively guide immunotherapy. Recently, a novel definition of cancer, integrating the dynamic crosstalk between malignant cells and immunocytes, has enabled the identification of novel immune-related subtypes associated with patient outcomes and response to ICIs [28]. Additionally, an immune-based classification system called the “immunoscore,” determined using T-cell infiltration, has been demonstrated to be more robust than tumor-node-metastasis classification [29]. In this study, we micro dissected gene expression profiles of patients with breast cancer and identified a new immune-related molecular classification system of breast cancer using the NMF algorithm. The established immune-related molecular subclasses were associated with response to ICIs, and predictors of the ICI response, such as TMB, NeoAgs, TILs, PD-1/PD-L1 expression, genomic alteration, and infiltrating immune cell types.

NMF, an analysis method that separates multiple-scale data into limited primary components, has been adopted in dissecting bulk sequencing data [30]. We divided patients into immune and non-immune classes based on the immunocyte infiltration level according to the NMF analysis. The patients in the immune class presented a higher immunocytes, immune activation-related signature, IFN-γ, cytolytic activation, and tertiary lymphoid structure, representing immune “hot” tumors. In contrast, the non-immune class represented immune “cold” tumors. These results were validated by probing the expression of markers of immunocytes using immunohistochemistry staining. Although immune “hot” tumors are infiltrated with immunocytes, immune-suppressive signaling may harness the antitumor immune effect [31, 32]. Therefore, investigating the components in the tumor immune niche is pivotal in cancer immunotherapy. According to stromal activation calculated using the NTP algorithm, the immune class was further separated into immune-activated and -suppressed subclasses. The immune-suppressed subclass showed higher scores for TGF-β signaling, such as fibroblast–TBRs, T-cell–TBRs, and late TGF-β signaling. TGF-β secreted by malignant epithelial cells, CAFs, and immunocytes further generate an immune-suppressive niche via metabolic reprogramming of the tumor and by orchestrating the inactivation of immune cells, leading to a decrease in the efficacy of anti-cancer immunotherapies [33]. Additionally, the immune-suppressed subclass was more enriched with immune suppressive cells and signaling (e.g., MDSCs, M2 macrophages, Treg cells, and PD-1 signaling) than the immune-activated subclass. These results were recapitulated in another four independent cohorts and validated through immunohistochemistry and immunofluorescence staining of clinical samples, confirming the robustness of our established molecular classification. Collectively, these results implied that patients belonging to the immune-activated subclass might respond to ICI monotherapy. In contrast, patients in the immune-suppressed subclass may need ICI in combination with agents to eliminate immune suppressive cells or molecules.

Immunotherapy has revolutionized the paradigm of cancer management, with promising and durable responses across various tumor types [12]. However, despite the identification of TMB, TILs, and PD-1 expression as markers to predict the response to ICIs, recognizing candidate patients who will respond to immunotherapy remains challenging [34]. To interrogate the predictive significance of the identified immune-related subclasses, we evaluated the response rate to ICIs of patients belonging to the three molecular subclasses. Unexpectedly, the immune-activated subclass had the highest response to ICIs, whereas the immune-suppressed subclass had the lowest response to ICIs. These results suggest that patients belonging to the immune-activated subclass may be potential candidates for ICI therapy, thus providing a new strategy for selecting patients to receive ICI therapy.

To explore the molecular characteristics from the perspective of genomics and transcriptomics, we investigated the CNAs, TMB, NeoAgs, and gene mutations among the immune-related subclasses. Recent studies have reported that patients with lower CNA burden show a better outcome and a favorable response to immunotherapy, which may be because higher CNA induces immune evasion [35]. Consistent with the findings of previous studies, lower CNA deletion at the arm level was observed in the immune class than in the non-immune class. Additionally, classic markers to predict the response to ICIs, such as TMB, NeoAgs, TIL, and PD-1/PD-L1, were significantly upregulated in the immune class compared with those in the non-immune class confirming susceptibility to immunotherapy in patients allocated to the immune class. Gene mutation landscape is a pivotal component involved in the varied immunophenotypes. We observed different mutation frequencies among the three subclasses, reflecting potential mechanisms influencing the tumor immune niche. Immune cell subtype analysis among the three subclasses also confirmed the immune-activated and -suppressed status of patients with breast cancer. Further experimental conformation is warranted for an in-depth investigation of the underlying molecular mechanisms. Additional validation in larger breast cancer cohorts receiving ICI therapy is also needed.

Tekpli et al. established a three-subclass classification with gradual levels of immune infiltration of breast cancer [36]. They also identified subclass B associated with a poor response to neoadjuvant chemotherapy and a pro-tumorigenic immune infiltration. This classification provides a novel prognostic factor of immune contexture, which may be applied to make precise treatment decisions and improve outcome of patients with breast cancer. Our study identified an immune-suppressive subclass, which shows significant characteristics of immune suppression. Thus, we add a novel immune-suppressive subclass to current molecular classification of breast cancer. Our results suggest that patients with breast cancer allocated to the immune-activated subclass have a “hot” immune status. The tumor may be regressed by ICI immunotherapy with single-agent treatment. In contrast, for patients in the immune-suppressed subclass, ICI therapy combined with a TGF-β inhibitor or an agent to eliminate immune-suppressive cells might improve efficacy. Our novel classification provides new insights and assists in identifying candidates for tailored optimal immunotherapy.

## MATERIALS AND METHODS

### Study cohort

The study cohort comprised 4184 patients with breast cancer from public databases whose omics data and the corresponding follow-up information were available. TCGA–Breast Cancer (BRCA) cohort, derived from UCSC Xena (http://xena.ucsc.edu/) and containing details of 1104 patients with breast cancer, was used as the training cohort. The validation cohort comprised four independent external data cohorts, which were obtained from the Gene Expression Omnibus (GSE2109 (*n* = 350), GSE25066 (*n* = 508), and GSE58644 (*n* = 318), http://www.ncbi.nlm.nih.gov/geo/), and Molecular Taxonomy of Breast Cancer International Consortium (METABRIC, *n* = 1904, https://www.cbioportal.org/). Clinical tumor samples from patients with breast cancer were obtained from West China Hospital. The trial was conducted in accordance with the Declaration of Helsinki (as revised in 2013). The study was approved by the Biomedical Ethics Committee of West China Hospital, and informed consent was obtained from all participants.

### Identification of the immune-related molecular subclasses in patients with breast cancer

As a gene expression profiler of the training cohort, an unsupervised NMF algorithm was used to conduct virtual microdissection [37]. To screen the immune-related NMF factor, the immune enrichment score of each patient was calculated using the ESTIMATE method [38]. The average expression value of the eighth pattern was significantly higher than that of other patterns. Thus, this pattern was regarded as an “immune factor”. The largest NMF decomposition weight among the remaining nine factors is selected as the representative of these nine factors, and then sorts the genes according to the difference between weight in factor 8 and max weight in other factors, and the top 150 genes are selected as “exemplar genes”. Then, we used NMFConsensus to dichotomize TCGA–BRCA cohort into immune and non-immune classes according to “exemplar genes”. To further correct the classification results from NMFConsensus, we used a multidimensional scaling random forest algorithm provided in the randomForest (v4.6-16) package. According to previously defined activated stroma signatures [39], the immune class was further allocated into immune-activated and immune-suppressed subclasses using the NTP (CMScaller_0.99.2 package) algorithm.

### Characterization of immunophenotypes of the established molecular subclasses

To characterize the immunophenotypes of the established molecular subclasses, gene set variation analysis (v1.34.0) package [40] and NTP algorithm were used to perform enrichment scoring and positive prediction of immune-related signatures established previously (Supplementary Table 2). For immune and non-immune classes, DEGs that met the following criteria were determined using DESeq2 software: padj < 0.05 and the absolute value of a log-2-fold change > 1. Functional enrichment analyses (Kyoto Encyclopedia of Genes and Genomes and Gene Ontology) of the DEGs were conducted using the clusterProfiler (v3.14.3) package [41]. GSEA was performed on molecular signature database gene sets (MSigDB) to enrich pathways in the immune class using the fgsea (v1.12.0) package [42].

CNAs, TMB, NeoAgs, and TILs were compared between the immune and non-immune subclasses. GDAC Firehose (https://gdac.broadinstitute.org) provided CNAs as calculated using GISTIC2.0 for use by researchers. Additionally, in a previous study, Saltz et al. [43] evaluated the abundance of TILs through hematoxylin and eosin-stained images of TCGA samples. The TMB was calculated using the maftools (v2.6.05) package based on TCGA–BRCA mutation data (http://xena.ucsc.edu/). NeoAg for individual patients in the training cohort was obtained from a previous study by Rooney et al. [44]. In addition, to identify genes with differential mutations among the three immunophenotypes, we first used the MutSigCV (v1.41) package to predict significant cancer-related mutated genes (*P* < 0.01) based on the mutation data, and then used independent tests to screen the differential mutation genes. Finally, we used maftools to display the mutation landscapes of the immunophenotypes.

### Validation of the robustness of established molecular classification

We used the top 150 DEGs between immune and non-immune classes in the training cohort as the classifier. The NMF and ESTIMATE algorithms were performed to dichotomize the validation cohorts into immune and non-immune classes. The NTP method was conducted to further divide the immune class into immune-activated or -suppressed subclasses.

### Prediction of response to immunotherapy of immune-related subclasses

To evaluate the response rate to ICIs of different molecular subclasses, TIDE (http://tide.dfci.harvard.edu/) algorithm and submap analyses (Genepattern module “submap”) were conducted.

### Assessment of tumor-infiltrating immune cell types

To explore the variations in immune cell types of different molecular subclasses, the CIBERSORT (https://cibersort.stanford.edu/) and TIMER (http://timer.cistrome.org/) analyses were performed.

### Immunohistochemistry and immunofluorescence

To verify the three immunophenotypes in patients with breast cancer, the tumor samples were subjected to immunohistochemistry and immunofluorescence staining. The primary antibodies used were anti-TGF-β1 (Invitrogen, MA516949), anti-PD-1 (Abcam, ab52587), anti-interferon (IFN)-γ (Abcam, ab231035), anti-CD8 (CST, 703065), anti-CD45 (Abcam, ab40763), anti-CD3 (Abcam, ab16669), anti-CD163 (Abcam, ab182422), anti-CD19 (Abcam, ab134114), and anti-granzyme B (CST, 468905). Details regarding these analyses are provided in a previous study [45].

### Statistical analysis

Statistical analyses were conducted using R software unless otherwise stated, and the statistical significance level was set at 0.05. Student’s *t*-test and analysis of variance were used to analyze normally distributed variables, and Wilcoxon and Kruskal–Wallis tests were used to analyze non-normally distributed variables. Fisher’s exact test and Pearson’s chi-squared test were conducted to evaluate categorical variables (ns (not significant): *P* > 0.05, ^*^P ≤ 0.05, ^**^P ≤ 0.01, ^***^P ≤ 0.001, or ^****^P ≤ 0.0001).

### Data availability

All data generated or analyzed during this study are included in this published article and its supplementary information files; further inquiries can be directed to the corresponding author.

## Supplementary Materials

Supplementary Figures

Supplementary Table 1

Supplementary Table 2
