# Supplementary material for: Establishment of a tumor immune microenvironment-based molecular classification system of breast cancer for immunotherapy
Source: Aging (Albany NY). 2021 Nov 11;13(21):24313–38. doi: 10.18632/aging.203682 (PMC8610112; doi:10.18632/aging.203682)
Supplement: Supplementary Figures [file aging-13-203682-s001.pdf]

SUPPLEMENTARY FIGURES

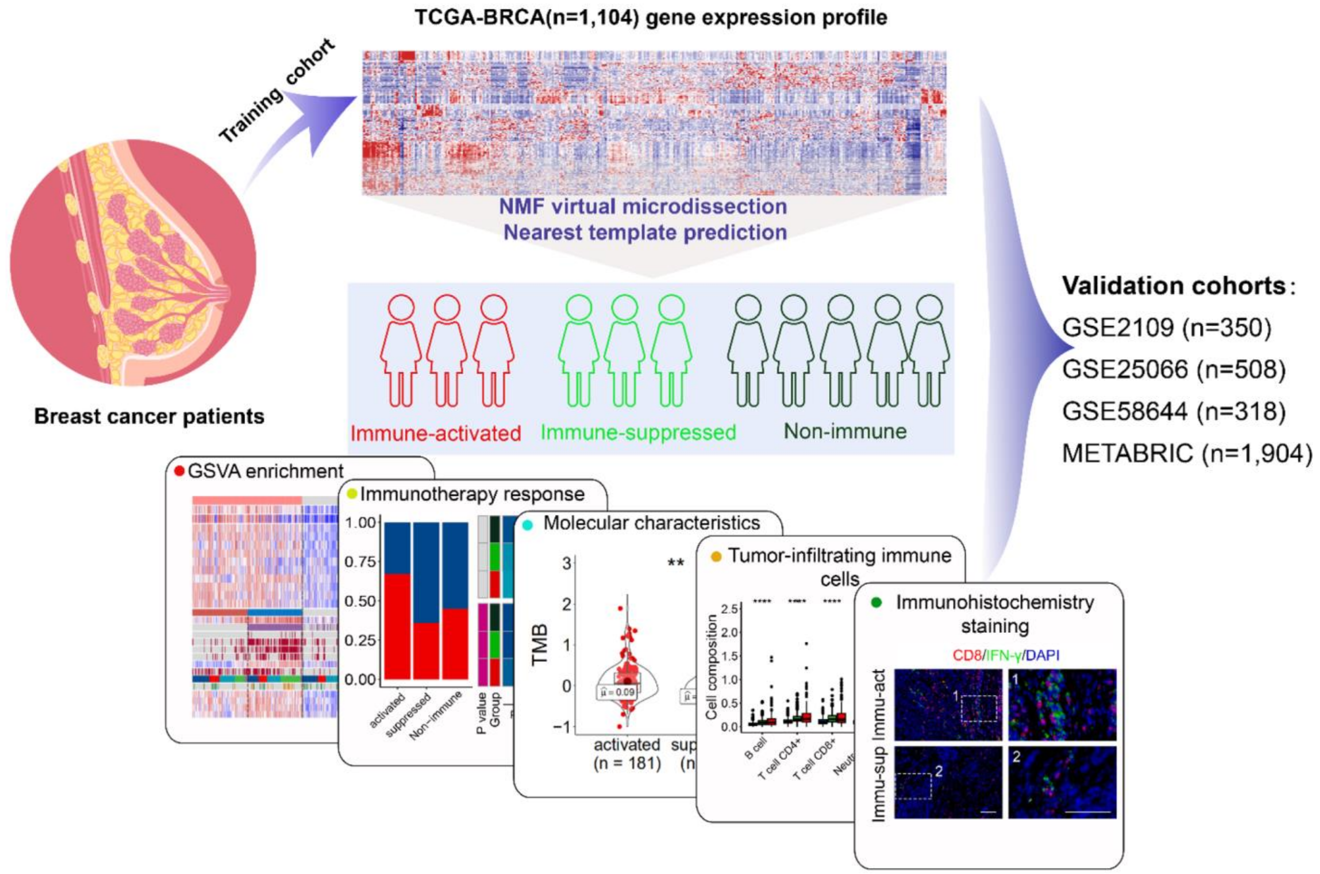

Supplementary Figure 1. Flowchart summarizing the analysis methods involved in this research.

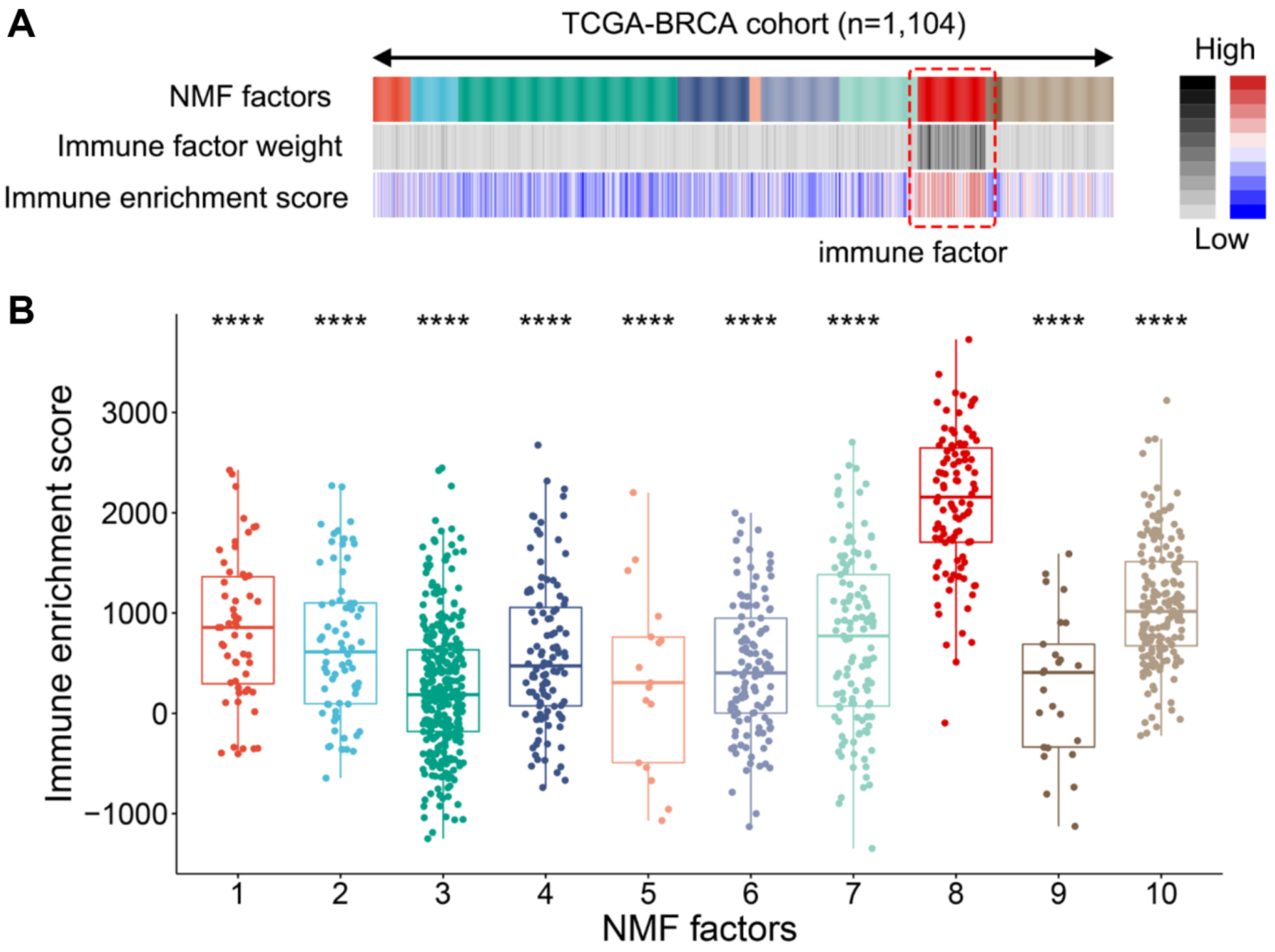

Supplementary Figure 2. "Immune factors" identified from bulk sequencing data. (**A**) A total of 10 patterns were identified using the NMF algorithm. Integration with the ESTIMATE algorithm revealed that the eighth pattern was enriched in most patients with high-immune enrichment scores. (**B**) Boxplots showed the immune enrichment score of each patient in different NMF factors. $^{****}P < 0.0001$. Abbreviation: NMF: non-negative matrix factorization.

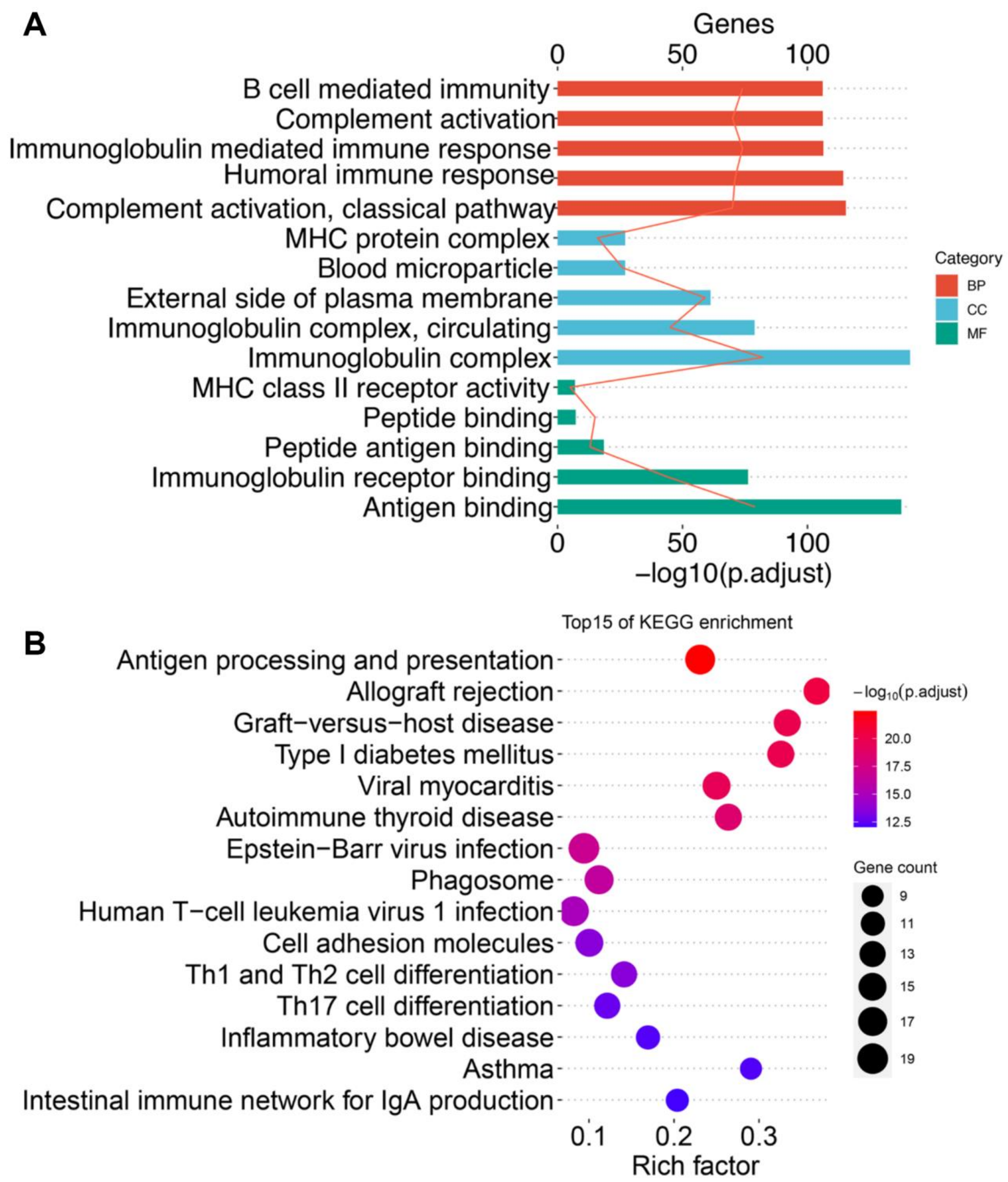

Supplementary Figure 3. Functional enrichment analysis of the top 150 exemplar genes in "immune factor." (**A**) Histogram showing the top 5 enriched pathways for each GO category. Different colors indicate different GO categories, the length of the column indicates the size of the adjust *p*-value, and the line indicates the number of enriched genes. (**B**) Bubble plot showing the top 15 enriched pathways for KEGG enrichment. The horizontal axis indicates the rich factor (number of enriched genes/number of genes contained in the pathway), the size of the dot indicates the number of genes enriched in each pathway, and the color of the dot indicates the adjust *p*-value. Abbreviations: GO: Gene Ontology; KEGG: Kyoto Encyclopedia of Genes.

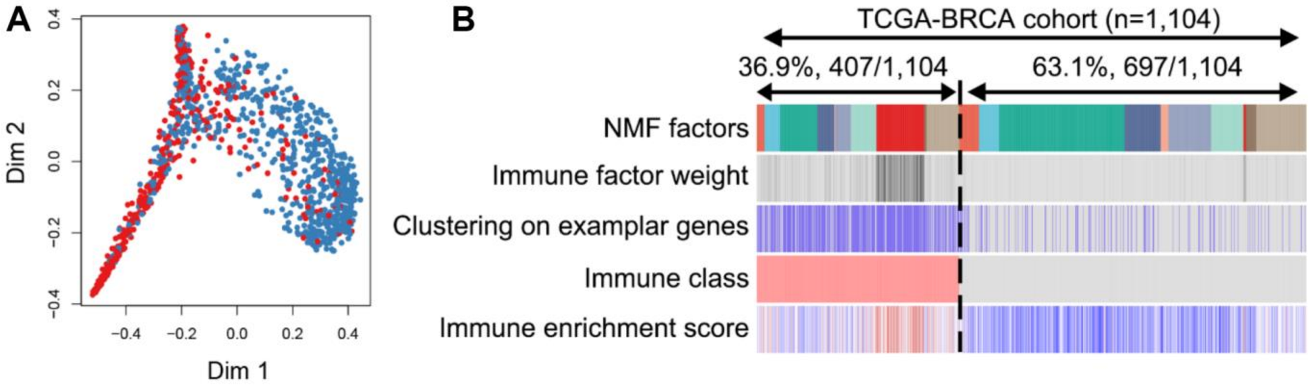

Supplementary Figure 4. Two immune-based classes were identified by consensus clustering. (**A**) The classification results of NMF consensus were further corrected using the multidimensional scaling random forest algorithm. (**B**) The heatmap shows the patients' distribution in different categories. Abbreviation: NMF: non-negative matrix factorization.

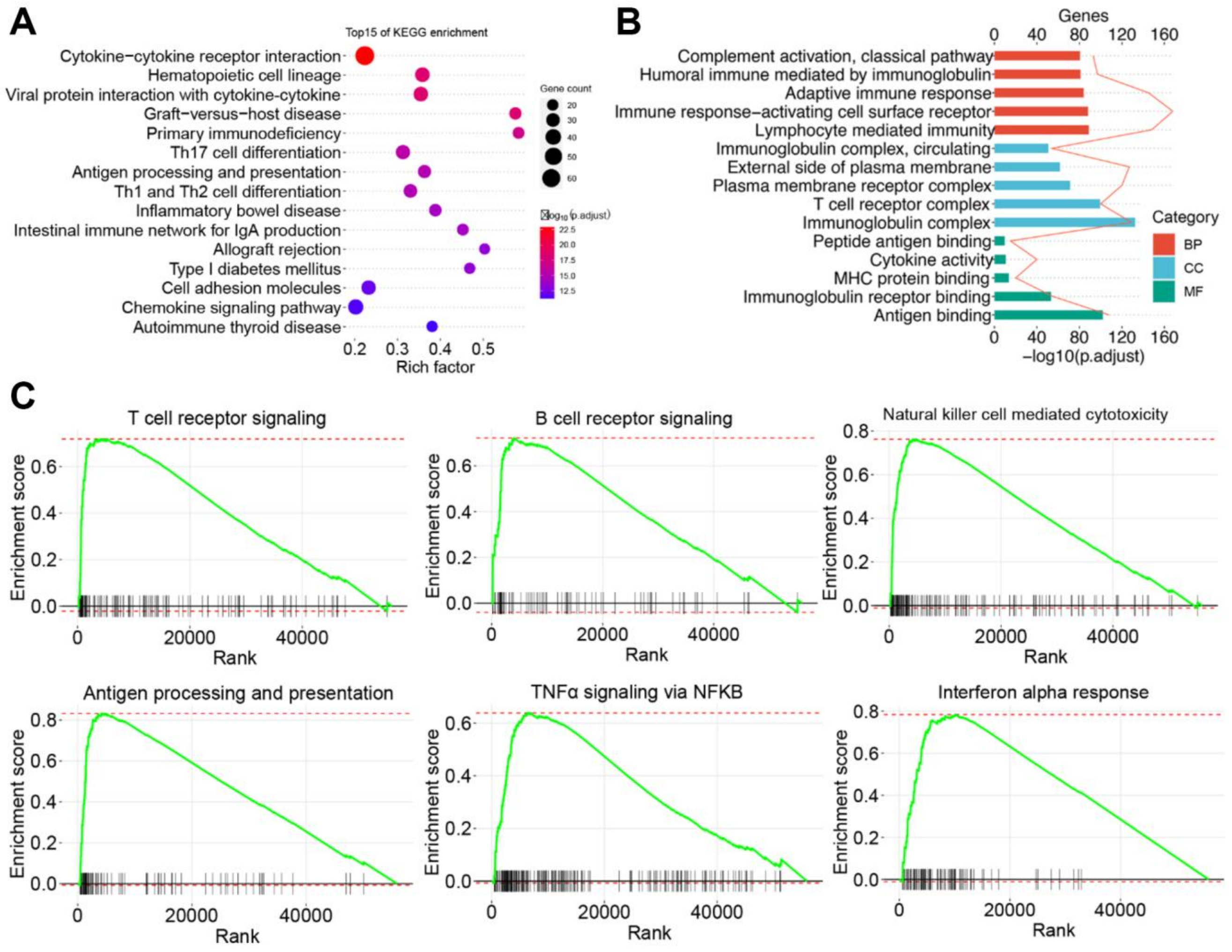

Supplementary Figure 5. Characteristics of immune activation for each immune class. (**A**) Bubble plot showing the top 15 enriched pathways for KEGG enrichment. The horizontal axis indicates the rich factor (number of enriched genes/number of genes contained in the pathway), the size of the dot indicates the number of genes enriched in each pathway, and the color of the dot indicates the size of the adjust *p*-value. (**B**) Histogram showing the top 5 enriched pathways of each GO category. Different colors indicate different GO categories, the length of the column indicates the size of the adjust *p*-value, and the line indicates the number of enriched genes. (**C**) Representative immune activation-related signaling pathways were evaluated by gene set enrichment analysis. Abbreviations: KEGG: Kyoto Encyclopedia of Genes; GO: Gene Ontology.

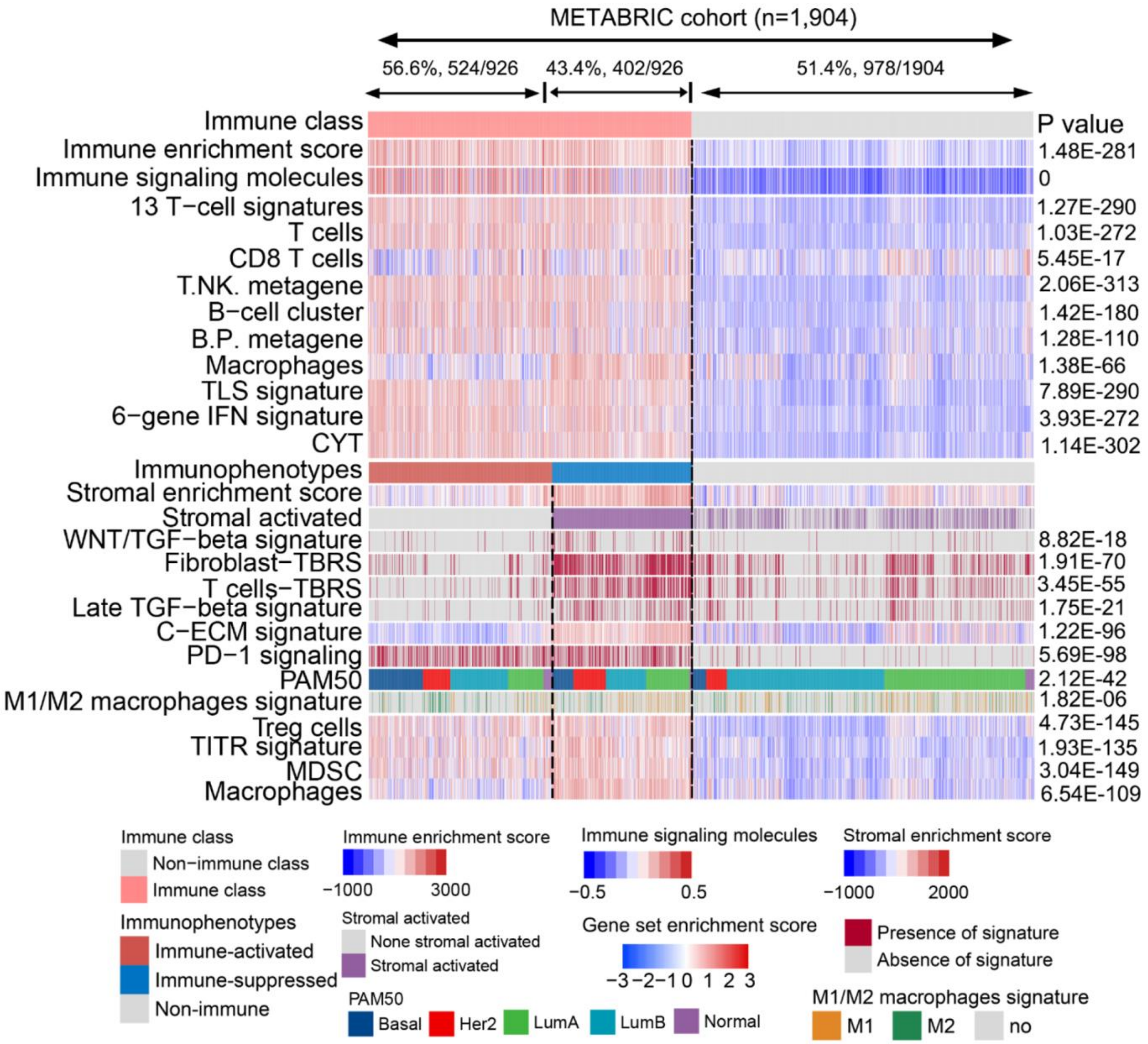

Supplementary Figure 6. Validation of the established immune-related molecular classification in the METABRIC cohort.

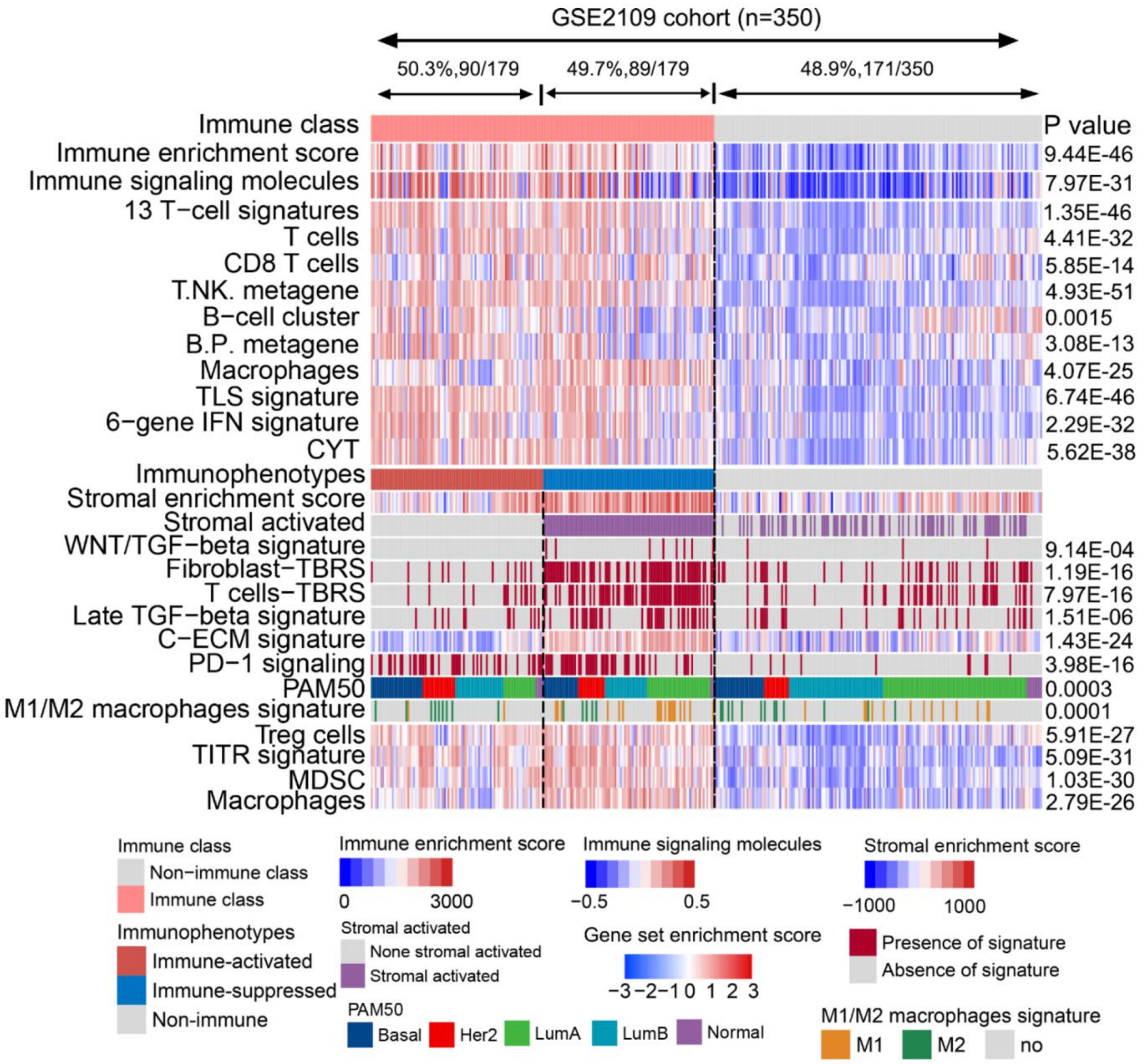

Supplementary Figure 7. Validation of the established immune-related molecular classification in the GSE2109 cohort.

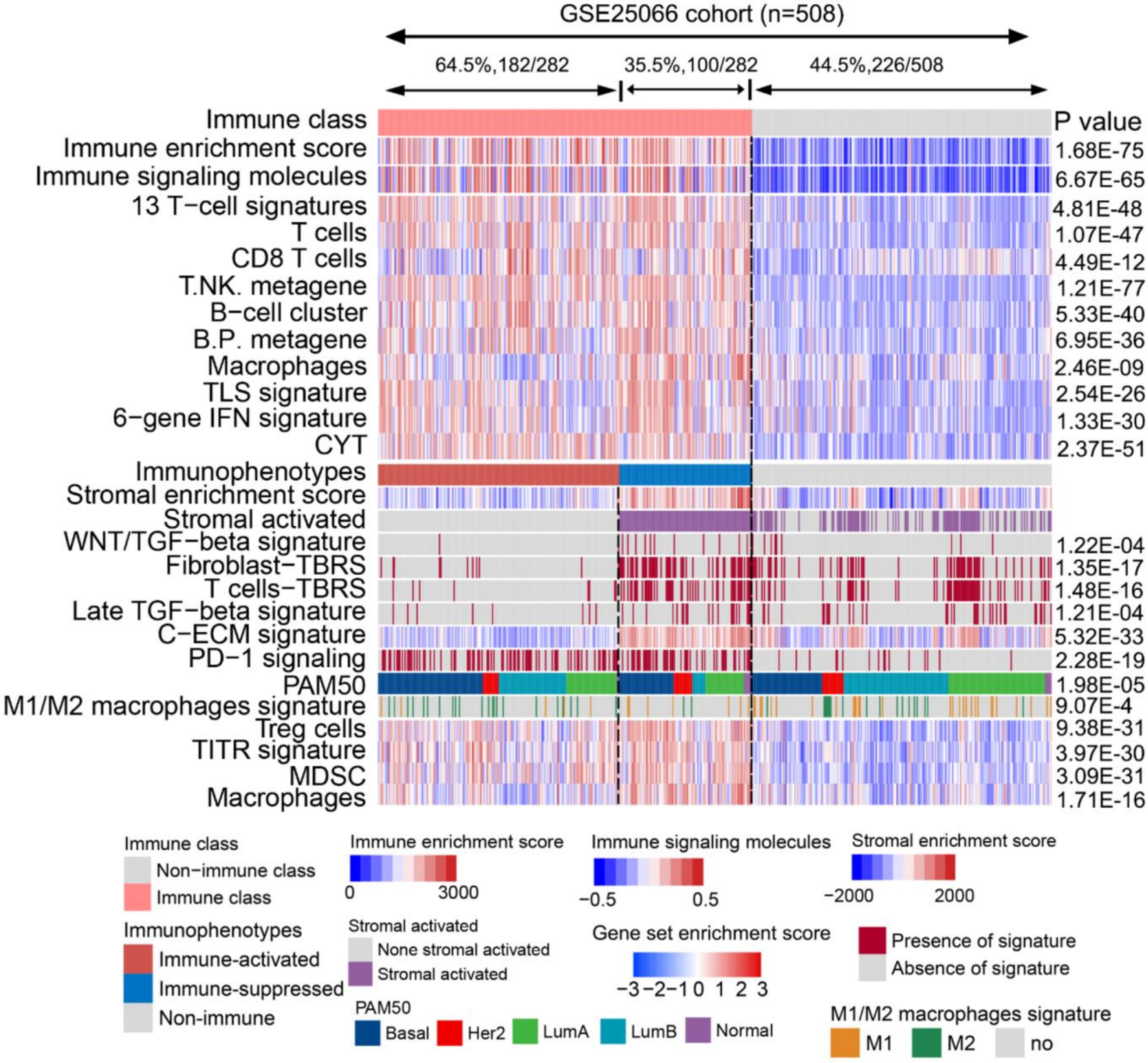

Supplementary Figure 8. Validation of the established immune-related molecular classification in the GSE25066 cohort.

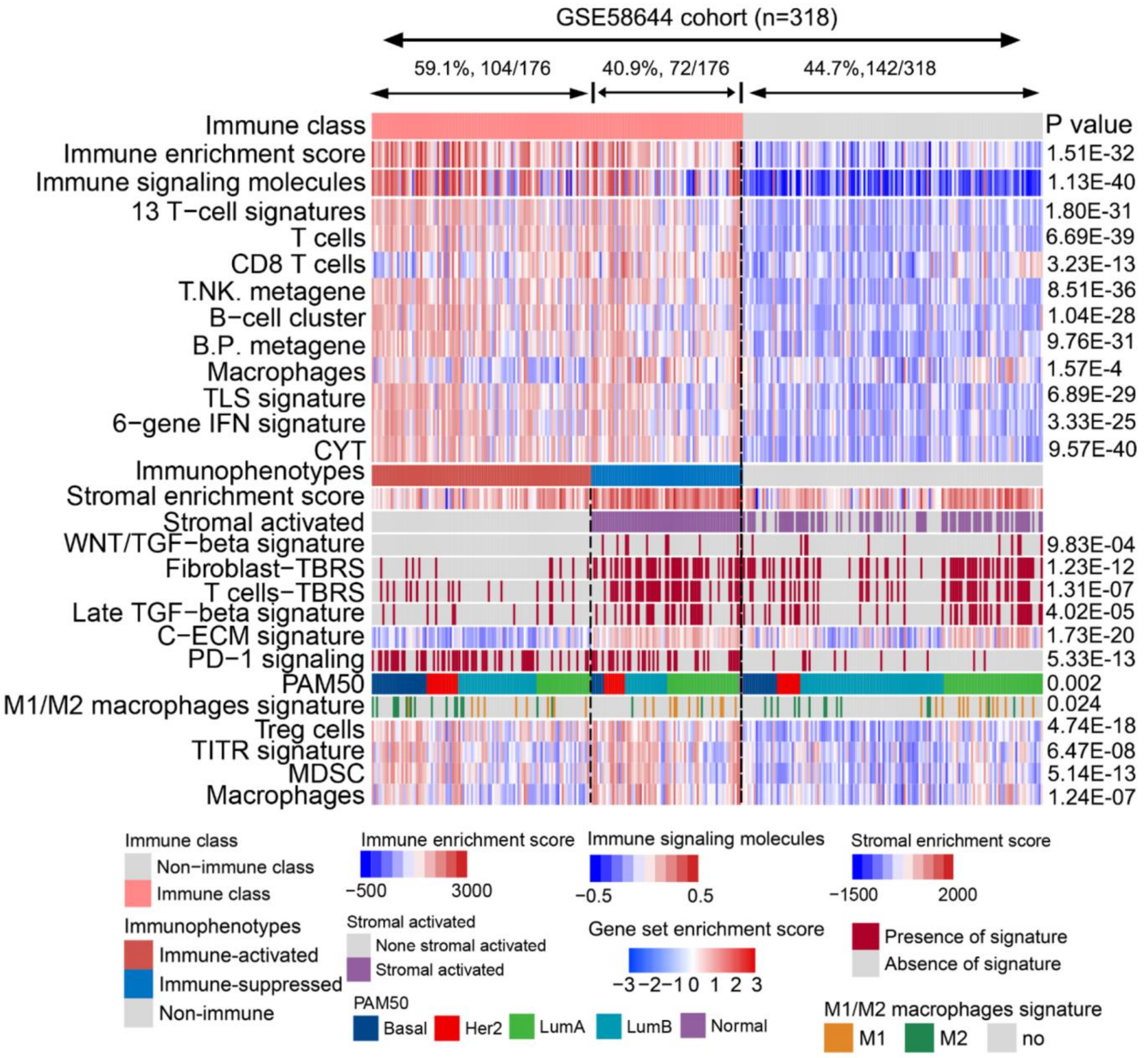

Supplementary Figure 9. Validation of the established immune-related molecular classification in the GSE58644 cohort.

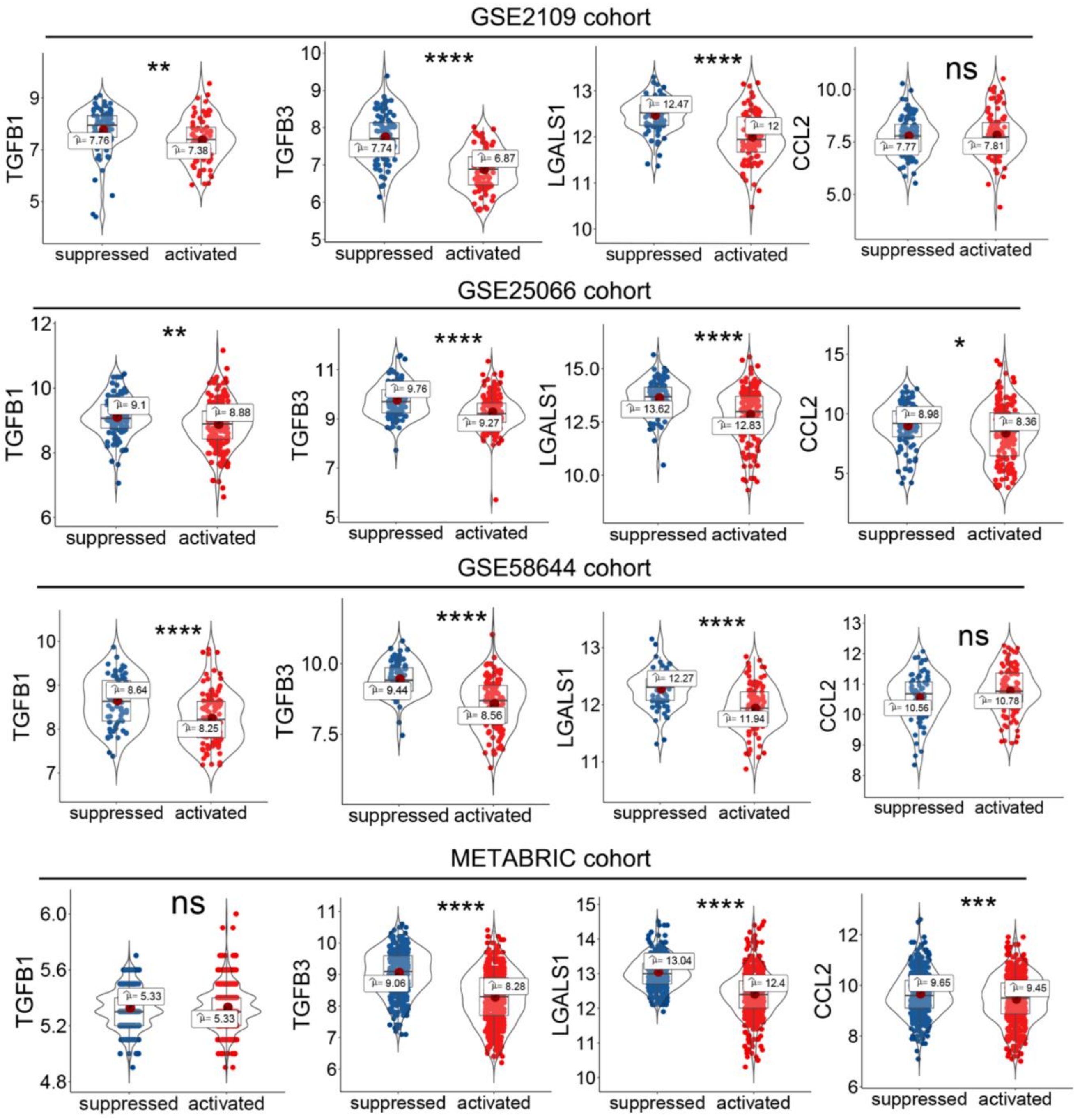

Supplementary Figure 10. Expression of immunosuppressive genes was compared between immune-activated and immune-suppressed subclasses in four independent external cohorts. ns: $P > 0.05$, $^{*}P \le 0.05$, $^{**}P \le 0.01$, $^{***}P \le 0.001$, or $^{****}P \le 0.0001$.
