# Supplementary material for: Establishment of a tumor immune microenvironment-based molecular classification system of breast cancer for immunotherapy
Source: Aging (Albany NY). 2021 Nov 11;13(21):24313–38. doi: 10.18632/aging.203682 (PMC8610112; doi:10.18632/aging.203682)
Supplement: Supplementary Table 1 [file aging-13-203682-s002.docx]

**Supplementary Table 1. Top 150 weighted genes of factor 8.**

|  | **Weight in factor 8** | **Max weight in other factors** | **Difference** |
| --- | --- | --- | --- |
| IGKC | 0.02535345 | 4.94e-324 | 0.02535345 |
| IGHG1 | 0.021517249 | 4.94e-324 | 0.021517249 |
| IGHA1 | 0.0197868 | 0.002871652 | 0.016915148 |
| IGLC2 | 0.01187528 | 4.94e-324 | 0.01187528 |
| IGKV3-20 | 0.009902792 | 2.57E-288 | 0.009902792 |
| IGHG2 | 0.009155825 | 1.96E-307 | 0.009155825 |
| IGLC3 | 0.00822123 | 4.94e-324 | 0.00822123 |
| IGHM | 0.008021188 | 4.94e-324 | 0.008021188 |
| IGKV4-1 | 0.007435028 | 4.94e-324 | 0.007435028 |
| IGLV2-14 | 0.007234587 | 4.94e-324 | 0.007234587 |
| IGKV1-5 | 0.00638215 | 4.94e-324 | 0.00638215 |
| CD74 | 0.012062736 | 0.00592842 | 0.006134316 |
| IGLV3-19 | 0.00550803 | 4.94e-324 | 0.00550803 |
| HLA-DRA | 0.011943581 | 0.006441327 | 0.005502254 |
| IGHV3-23 | 0.005264491 | 4.94e-324 | 0.005264491 |
| IGKV3-11 | 0.004975501 | 1.48219693752374e-323 | 0.004975501 |
| IGHG4 | 0.004919435 | 0.000177737 | 0.004741698 |
| B2M | 0.008914196 | 0.004239694 | 0.004674501 |
| IGHV1-18 | 0.004435588 | 4.94e-324 | 0.004435588 |
| IGHG3 | 0.004235719 | 6.71E-298 | 0.004235719 |
| IGLV3-21 | 0.004161134 | 4.94e-324 | 0.004161134 |
| IGLV1-51 | 0.004070293 | 4.94e-324 | 0.004070293 |
| IGLV3-25 | 0.004057138 | 4.94e-324 | 0.004057138 |
| IGLV1-40 | 0.004043295 | 2.43E-227 | 0.004043295 |
| IGHV4-39 | 0.003733977 | 4.94e-324 | 0.003733977 |
| IGLV2-23 | 0.003626316 | 6.73E-274 | 0.003626316 |
| IGHV5-51 | 0.00358488 | 4.94e-324 | 0.00358488 |
| IGJ | 0.003537641 | 0.000485708 | 0.003051933 |
| HLA-DRB1 | 0.005905054 | 0.00291349 | 0.002991564 |
| IGLV1-44 | 0.002891342 | 4.94e-324 | 0.002891342 |
| HLA-B | 0.008436413 | 0.005918743 | 0.002517671 |
| IGLV1-47 | 0.002441753 | 4.94e-324 | 0.002441753 |
| IGLV2-11 | 0.002437473 | 4.73E-307 | 0.002437473 |
| IGLV3-1 | 0.002407248 | 4.94e-324 | 0.002407248 |
| IGHV3-15 | 0.002265696 | 4.94e-324 | 0.002265696 |
| IGHV3-21 | 0.002152948 | 6.83E-300 | 0.002152948 |
| LYZ | 0.00268586 | 0.000547445 | 0.002138415 |
| IGHV4-59 | 0.002100261 | 4.94e-324 | 0.002100261 |
| IGHV3-30 | 0.002077693 | 4.94e-324 | 0.002077693 |
| IGKV3-15 | 0.002070889 | 4.94e-324 | 0.002070889 |
| IGHA2 | 0.003039799 | 0.001174635 | 0.001865164 |
| IGLV6-57 | 0.001822955 | 4.94e-324 | 0.001822955 |
| IGHV3-11 | 0.001748408 | 4.94e-324 | 0.001748408 |
| IGLV3-10 | 0.001730443 | 4.94e-324 | 0.001730443 |
| IGHV1-2 | 0.00163919 | 9.06E-305 | 0.00163919 |
| IGLV4-69 | 0.00159819 | 4.94e-324 | 0.00159819 |
| CXCL9 | 0.001530376 | 2.91E-34 | 0.001530376 |
| IGHV1-69-2 | 0.001525 | 4.94e-324 | 0.001525 |
| HLA-E | 0.003045069 | 0.001561466 | 0.001483603 |
| IGKV1-9 | 0.001402741 | 8.08E-179 | 0.001402741 |
| IGKV1-17 | 0.001382303 | 4.94e-324 | 0.001382303 |
| IGHV3-73 | 0.001345876 | 4.94e-324 | 0.001345876 |
| IGLV8-61 | 0.001313523 | 4.94e-324 | 0.001313523 |
| IGHV3-33 | 0.001257705 | 4.94e-324 | 0.001257705 |
| IGKV1-16 | 0.001198802 | 4.10E-214 | 0.001198802 |
| IGHV1-24 | 0.001168643 | 2.52E-76 | 0.001168643 |
| IGHV3-49 | 0.001148075 | 4.94e-324 | 0.001148075 |
| IGHV4-34 | 0.001100834 | 4.65E-212 | 0.001100834 |
| CD52 | 0.00110316 | 3.37E-05 | 0.001069413 |
| IGHV3-74 | 0.001051744 | 1.88E-222 | 0.001051744 |
| IGHGP | 0.001044705 | 5.66130060943651e-319 | 0.001044705 |
| IGLV2-8 | 0.001031611 | 3.38E-220 | 0.001031611 |
| IGHV1-46 | 0.001029229 | 4.03E-296 | 0.001029229 |
| HLA-DRB5 | 0.002090054 | 0.001130094 | 0.00095996 |
| C1QB | 0.001453743 | 0.000513445 | 0.000940298 |
| IGKV1-27 | 0.000937607 | 4.94e-324 | 0.000937607 |
| IGKV1-6 | 0.000893125 | 2.18E-171 | 0.000893125 |
| CCL19 | 0.00093835 | 5.27E-05 | 0.000885608 |
| IGLL5 | 0.000836289 | 4.94e-324 | 0.000836289 |
| CCL5 | 0.000946595 | 0.000116527 | 0.000830067 |
| C1QA | 0.00150895 | 0.000723286 | 0.000785665 |
| IGHV4-31 | 0.000744725 | 1.38599599526512e-309 | 0.000744725 |
| C1QC | 0.001356676 | 0.000631124 | 0.000725552 |
| HLA-DPB1 | 0.001614292 | 0.000900182 | 0.00071411 |
| IGHV2-26 | 0.000697363 | 4.94e-324 | 0.000697363 |
| IGHJ3 | 0.000696212 | 4.94e-324 | 0.000696212 |
| IGKV2-24 | 0.000682733 | 2.05E-251 | 0.000682733 |
| IGHV3-48 | 0.000633607 | 9.59E-227 | 0.000633607 |
| STAT1 | 0.000993328 | 0.000408682 | 0.000584646 |
| IGLV7-46 | 0.000584592 | 1.21E-40 | 0.000584592 |
| IGHV1-69 | 0.000576334 | 4.94e-324 | 0.000576334 |
| IGLV3-9 | 0.000561334 | 9.14E-84 | 0.000561334 |
| HLA-A | 0.004814253 | 0.004289393 | 0.00052486 |
| IGHV2-70 | 0.000522151 | 4.94e-324 | 0.000522151 |
| IGLV7-43 | 0.000517899 | 5.32E-293 | 0.000517899 |
| IGLV3-27 | 0.000508517 | 1.23E-197 | 0.000508517 |
| IGHV3-72 | 0.000500665 | 2.37E-223 | 0.000500665 |
| IGKV2D-29 | 0.000470353 | 5.08E-197 | 0.000470353 |
| IGLC7 | 0.000457235 | 1.52E-147 | 0.000457235 |
| IGLV5-45 | 0.000452621 | 2.14E-173 | 0.000452621 |
| IGHV2-5 | 0.00044292 | 4.94e-324 | 0.00044292 |
| IGHV3-53 | 0.000436099 | 3.59E-143 | 0.000436099 |
| IGLV1-36 | 0.000403837 | 4.94e-324 | 0.000403837 |
| TRAC | 0.000489645 | 9.11E-05 | 0.000398553 |
| IGKV3D-20 | 0.000391095 | 6.26E-241 | 0.000391095 |
| IGHV3-13 | 0.000373417 | 7.75E-154 | 0.000373417 |
| SRGN | 0.000949775 | 0.000582451 | 0.000367325 |
| PIM2 | 0.000381019 | 1.48E-05 | 0.000366202 |
| HLA-DQB1 | 0.000628133 | 0.000262717 | 0.000365417 |
| AC244250.2 | 0.000370884 | 7.54E-06 | 0.000363341 |
| IL2RG | 0.000387748 | 2.55E-05 | 0.000362212 |
| TRBC2 | 0.000382421 | 3.06E-05 | 0.000351841 |
| IGLV4-60 | 0.000346482 | 8.48E-240 | 0.000346482 |
| CD79A | 0.00034392 | 4.94e-324 | 0.00034392 |
| TAP1 | 0.000666545 | 0.000330144 | 0.000336402 |
| HLA-DQA1 | 0.000524372 | 0.000205047 | 0.000319325 |
| HLA-DPA1 | 0.000975444 | 0.00066137 | 0.000314073 |
| MZB1 | 0.000372463 | 6.91E-05 | 0.000303404 |
| CTSS | 0.00050965 | 0.000209963 | 0.000299688 |
| LCP1 | 0.000573405 | 0.000279803 | 0.000293603 |
| CD2 | 0.00033503 | 4.20E-05 | 0.000293007 |
| CD53 | 0.000483955 | 0.000195908 | 0.000288047 |
| RAC2 | 0.000383006 | 9.97E-05 | 0.000283356 |
| NKG7 | 0.000294101 | 1.25E-05 | 0.000281595 |
| HLA-DQA2 | 0.000467615 | 0.000186798 | 0.000280817 |
| IGHJ2 | 0.000279139 | 3.53E-274 | 0.000279139 |
| IGHV3-43 | 0.000276972 | 1.93E-141 | 0.000276972 |
| LTB | 0.000275227 | 6.10E-07 | 0.000274616 |
| IGLV2-18 | 0.000270238 | 8.47E-277 | 0.000270238 |
| CXCR4 | 0.000590252 | 0.000335355 | 0.000254897 |
| HLA-DMA | 0.000514965 | 0.000272831 | 0.000242133 |
| GBP4 | 0.000274771 | 3.40E-05 | 0.000240794 |
| CD3D | 0.000254384 | 1.75E-05 | 0.000236903 |
| IGLV10-54 | 0.000250744 | 1.51E-05 | 0.000235659 |
| IGHD | 0.000233923 | 8.01E-51 | 0.000233923 |
| CORO1A | 0.000284491 | 5.32E-05 | 0.000231267 |
| IGHV3-66 | 0.000222525 | 2.14E-250 | 0.000222525 |
| APOL1 | 0.000426305 | 0.000208226 | 0.000218079 |
| GZMA | 0.000248507 | 3.18E-05 | 0.000216749 |
| IGHV4-28 | 0.000216486 | 2.73E-195 | 0.000216486 |
| IGHV4-61 | 0.000209982 | 1.89E-305 | 0.000209982 |
| IGKV2-30 | 0.000201171 | 1.05E-292 | 0.000201171 |
| CD3E | 0.000224221 | 2.33E-05 | 0.000200957 |
| HLA-DRB6 | 0.000340586 | 0.000144761 | 0.000195825 |
| APOC1 | 0.000615624 | 0.000422981 | 0.000192643 |
| IGKJ5 | 0.000184142 | 1.12E-06 | 0.000183024 |
| TRBV28 | 0.000193543 | 1.29E-05 | 0.000180679 |
| LAP3 | 0.000370312 | 0.000204806 | 0.000165506 |
| HLA-C | 0.005145041 | 0.00498028 | 0.000164761 |
| SLAMF7 | 0.000159512 | 1.08E-42 | 0.000159512 |
| PTGDS | 0.000239238 | 8.06E-05 | 0.000158668 |
| IGHV3-7 | 0.000158521 | 2.43E-223 | 0.000158521 |
| CD27 | 0.000154301 | 1.06E-06 | 0.000153238 |
| LAPTM5 | 0.00098999 | 0.000840377 | 0.000149612 |
| DERL3 | 0.00016436 | 1.75E-05 | 0.00014683 |
| HLA-F | 0.000298506 | 0.000151978 | 0.000146529 |
| GBP1 | 0.000365074 | 0.000218903 | 0.00014617 |
| CCL21 | 0.000300398 | 0.000154487 | 0.000145911 |
| PSMB9 | 0.000268475 | 0.000123381 | 0.000145094 |
| HCP5 | 0.000228318 | 8.36E-05 | 0.000144668 |
