# Supplementary material for: Establishment of a tumor immune microenvironment-based molecular classification system of breast cancer for immunotherapy
Source: Aging (Albany NY). 2021 Nov 11;13(21):24313–38. doi: 10.18632/aging.203682 (PMC8610112; doi:10.18632/aging.203682)
Supplement: Supplementary Table 2 [file aging-13-203682-s003.pdf]

SUPPLEMENTARY TABLE

Supplementary Table 2. Previously established immune-related signatures.

Signature name	Reference
Immune enrichment score	Yoshihara et al. Nat Commun. 2013; 4:2612
Stromal enrichment score	Yoshihara et al. Nat Commun. 2013; 4:2612
Immune signaling molecules	Cancer Genome Atlas Network. Cell. 2015; 161:1681–96
13 T-cell signature	Spranger et al. Proc Natl Acad Sci U S A. 2016; 113:E7759–68.
T cells	Bindea et al. Immunity. 2013; 39:782–95
CD8 T cells	Bindea et al. Immunity. 2013; 39:782–95
Treg cells	Angelova et al. Genome Biol. 2015; 16:64
TITR signature	Magnuson et al. Proc Natl Acad Sci U S A. 2018; 115:E10672-e81
MDSC	Angelova et al. Genome Biol. 2015; 16:64
T.NK. metagene	Alistar et al. Genome Med. 2014; 6:80
B-cell cluster	Iglesia et al. Clin Cancer Res. 2014; 20:3818–29.
B.P. metagene	Alistar et al. Genome Med. 2014; 6:80
Macrophages	Bindea et al. Immunity. 2013; 39:782–95
TLS signature	Finkin et al. Nat Immunol. 2015; 16:1235–44
6-gene IFN signature	Chow et al. J Clin Oncol. 2016; 34:318–327
CYT	Rooney et al. Cell. 2015; 160:48–61
WNT/TGF-β signature	Lachenmayer et al. Clin Cancer Res. 2012; 18:4997–5007
C-ECM signature	Chakravarthy et al. Nat Commun. 2018; 9:4692
M1/M2 macrophages	Coates PJ et al. Cancer Res. 2008; 68:450–6
Late TGFB signature	Coulouarn C et al. Hepatology. 2008; 47:2059–67
T cells-TBRS	Calon A et al. Cancer Cell. 2012; 22:571–84
Fibroblast-TBRS	Calon A et al. Cancer Cell. 2012; 22:571–84
Stromal activated	Moffitt RA et al. Nat Genet. 2015; 47:1168-78
PD-1 signaling	Quigley M et al. Nat Med. 2010; 16:1147–51
IS	Thorsson V et al. Immunity. 2018; 48:812e830.e14.
PAM50	Parker et al. J Clin Oncol. 2009; 27.8:1160

Abbreviations: TITR: tumor-infiltrating Tregs; MDSC: myeloid-derived suppressor cell; IFN: interferon; TLS: tertiary lymphoid structure; CYT: cytolytic activity score; C-ECM: cancer-associated extracellular matrix.
